# Porcine Adenovirus-Based Viral Vectors for Swine Vaccines: A Scoping Review and Future Perspectives

**DOI:** 10.3390/ani16203247

**Published:** 2026-10-15

**Authors:** Nattawooti Sthitmatee, Karnrawee Suanpat, Yupa Sington, Anucha Muenthaisong, Kanokwan Sangkakam, Boondarika Nambooppha, Amarin Rittipornlertrak, Nisachon Apinda, Pongpisid Koonyosying, Thanya Varinrak, Khwanchai Kreausukon

**Affiliations:** 1 Laboratory of Veterinary Vaccine and Biological Products, Faculty of Veterinary Medicine, Chiang Mai University, Chiang Mai 50100, Thailand; karnrawee_suanpat@cmu.ac.th (K.S.); yupa_sing@cmu.ac.th (Y.S.); pharham16500@gmail.com (A.M.); kanokwansangkakam@gmail.com (K.S.); boondarika.n@cmu.ac.th (B.N.); amarin.r@cmu.ac.th (A.R.); nisachon.a@cmu.ac.th (N.A.); pongpisid_koo@nation.ac.th (P.K.); thanya.var@cmu.ac.th (T.V.); khwanchai.kreau@cmu.ac.th (K.K.); 2 Faculty of Medicine, Nation University, San Sai, Chiang Mai 50210, Thailand; 3 Veterinary Diagnostic and Technology Transfer Center, Faculty of Veterinary Medicine, Chiang Mai University, Chiang Mai 50100, Thailand

**Keywords:** mucosal immunity, porcine adenovirus, swine vaccine, viral vector, veterinary vaccine development

## Abstract

Porcine adenoviruses (PAdVs) are promising but still underdeveloped viral-vector platforms for swine vaccine development. Compared with human adenovirus-based vectors, PAdV vectors may offer potential advantages in host association, veterinary relevance, and mucosal delivery while avoiding direct reliance on common human adenovirus backbones. This review summarizes current knowledge on PAdV biology, vector engineering, vaccine applications, immune responses, translational challenges, and future priorities. Early studies showed that recombinant PAdV vectors can express heterologous antigens from important swine pathogens, including classical swine fever virus, pseudorabies virus, and transmissible gastroenteritis virus. The strongest direct proof-of-concept evidence comes from recombinant PAdV expressing classical swine fever virus E2/gp55, which induced neutralizing antibodies and protection in pigs. Comparative adenovirus-vectored swine vaccine studies support the broader feasibility of adenoviral antigen delivery for porcine reproductive and respiratory syndrome, porcine epidemic diarrhea, swine influenza, and African swine fever, but these data should not be interpreted as direct validation of PAdV for these diseases. Major gaps remain in anti-PAdV immunity, genetic stability, biosafety, shedding, target-animal safety, DIVA compatibility, manufacturing, and regulatory readiness. Recent advances in PAdV-5 reverse genetics provide new opportunities for modern vector design.

## Simple Summary

Swine diseases cause major health and economic losses worldwide, and improved vaccine platforms are needed to control both established and emerging infections. Porcine adenoviruses (PAdVs) naturally infect pigs and can be engineered to carry genes encoding protective antigens from other pathogens. This review summarizes current knowledge on PAdV-based vaccine vectors, including their biology, genetic engineering, immune responses, potential applications, and remaining challenges. Early studies showed that recombinant PAdVs can express antigens from classical swine fever virus, pseudorabies virus, and transmissible gastroenteritis virus. The strongest direct evidence comes from classical swine fever studies, in which a PAdV vector expressing the E2/gp55 antigen induced neutralizing antibodies and protection in pigs. Evidence from other adenovirus-vectored vaccines suggests possible design principles for porcine reproductive and respiratory syndrome, porcine epidemic diarrhea, swine influenza, and African swine fever, but these findings remain mostly comparative rather than direct PAdV evidence. Important issues, including pre-existing anti-PAdV immunity, safety, shedding, genetic stability, manufacturing, and regulatory approval, must be addressed before practical use. Recent advances in PAdV-5 reverse genetics provide new opportunities to improve this platform for future swine vaccine research.

## 1. Introduction

Swine production is essential to global animal agriculture and food security, but it remains threatened by viral diseases that cause economic losses, impair animal welfare, and disrupt trade, including classical swine fever (CSF), Aujeszky’s disease (AD), porcine reproductive and respiratory syndrome (PRRS), porcine epidemic diarrhea (PED), transmissible gastroenteritis (TGE), swine influenza (SI), and African swine fever (ASF). Although live attenuated and inactivated vaccines have contributed to disease control, they may be limited by safety concerns, incomplete cross-protection, maternal antibody interference, weak mucosal immunity, difficulty differentiating infected from vaccinated animals, and slow adaptation to emerging variants [1,2,3,4]. These limitations have encouraged the development of next-generation vaccine platforms that enable rational antigen design, rapid genetic modification, and induction of systemic and mucosal immunity.

Viral vector vaccines are widely explored in modern vaccinology, and adenoviral vectors are particularly attractive because they efficiently deliver foreign genes, support strong antigen expression, infect dividing and non-dividing cells, and stimulate innate and adaptive immune responses. As non-enveloped, double-stranded DNA viruses with relatively stable genomes, adenoviruses are suitable for genetic manipulation and vaccine delivery. They can promote antigen presentation, antibody production, and T-cell-mediated immunity, which are important for protection against many viral infections [5,6,7,8]. Human adenovirus 5 (HAdV-5) has been widely used as a prototype vector because it is well characterized and supported by established production systems. However, pre-existing anti-vector immunity can reduce vector transduction, antigen expression, and vaccine efficacy, particularly after repeated administration [9,10]. This limitation has increased interest in rare HAdV and non-HAdV vectors, including chimpanzee adenoviruses (ChAdV), bovine adenoviruses (BAdV), canine adenoviruses (CAdV), fowl adenoviruses (FAdV), and porcine adenoviruses (PAdV), as alternative vaccine platforms [5,11,12].

PAdVs are especially relevant for swine vaccine development because they originate from pigs and may provide a species-associated vector backbone for antigen delivery in the target host. Foundational studies on porcine adenovirus 3 (PAdV-3), including its complete genome sequence and transcriptional map, established the basis for understanding genome organization and identifying regions suitable for genetic manipulation [13]. Subsequent studies showed that PAdV-3 could be engineered as an expression vector and that E1-deleted recombinant PAdV-3 vectors could support heterologous gene expression [14,15]. Porcine adenovirus 5 (PAdV-5) has also been explored as a vector platform. Recombinant PAdV-5 expressing the TGEV spike gene was constructed and characterized [16], while recent molecular characterization, isolation, and reverse-genetic manipulation of PAdV-5 have renewed interest in this serotype as an emerging vaccine platform [17,18].

Experimental studies have provided early proof-of-concept evidence that PAdV vectors can deliver protective antigens and stimulate immune responses in pigs. Recombinant PAdV expressing CSFV gp55/E2 induced immune responses and protected pigs against challenge in selected models [19,20,21]. A DNA prime followed by recombinant PAdV boost also protected pigs against CSF, supporting the use of PAdV in heterologous vaccination regimens [22]. In addition, recombinant PAdV expressing PRV glycoprotein D (gD) and PAdV-5 expressing TGEV spike demonstrated that PAdV vectors can deliver antigens from different swine pathogens [16,23].

Broader adenovirus-vectored vaccine studies in pigs provide useful comparative evidence for evaluating PAdV. Recombinant adenoviral vectors have been investigated for SI, PRRS, PED, and ASF [24,25,26,27,28,29,30,31], showing that adenoviral platforms can induce antigen-specific antibodies, IFN-γ responses, cytotoxic T-cell responses, and protection in some models. However, strong immunogenicity does not always result in complete protection, particularly for complex pathogens such as ASFV and PRRSV [32,33]. Therefore, successful PAdV-based vaccines will require an appropriate vector backbone, rational antigen selection, optimized delivery routes, and disease-specific immune evaluation.

Despite its promise, PAdV vector development remains less advanced than HAdV, ChAdV, and other non-HAdV platforms. Available studies are limited and heterogeneous in vector serotype, deletion strategy, antigen insert, administration route, animal model, immune endpoints, and challenge design. Key translational issues remain unresolved, including pre-existing immunity to PAdV in commercial pigs, genetic stability, producer cell systems, vector shedding, recombination risk, manufacturing scalability, and regulatory feasibility [5,17,18,34]. A scoping review is therefore appropriate to broadly map evidence from virus biology, vector engineering, antigen expression, experimental vaccination, immunological evaluation, and comparative adenoviral-vector research.

**Figure 1:**
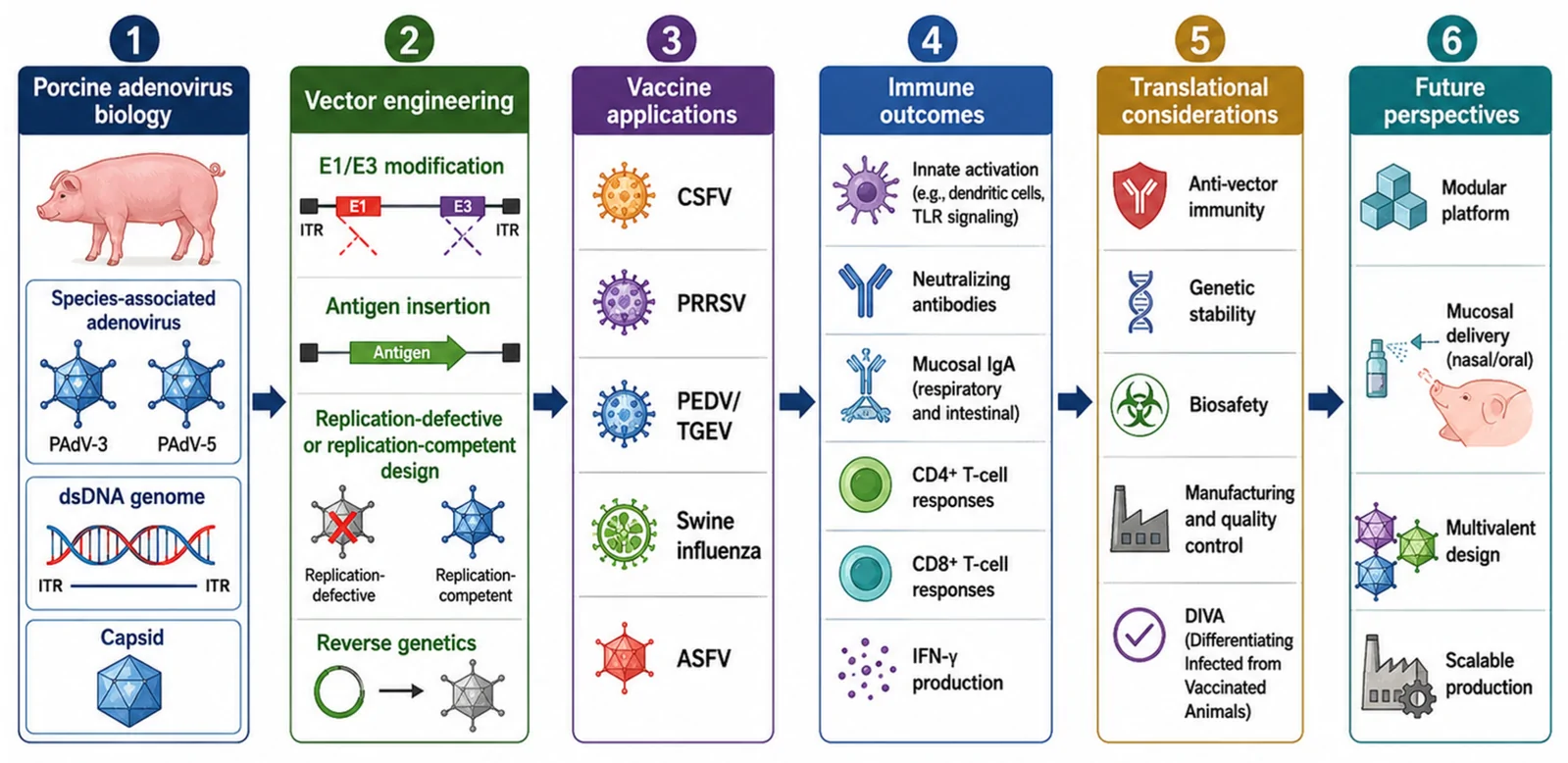
Conceptual framework of porcine adenovirus-based viral vectors for swine vaccines. This figure summarizes the overall framework of PAdV-based vaccine-vector development, beginning with PAdV biology and candidate backbones, followed by vector engineering, vaccine applications, immune outcomes, translational considerations, and future development priorities. PAdV-3 and PAdV-5 are highlighted as candidate swine-associated adenoviral backbones that can be engineered for heterologous antigen delivery. The framework emphasizes the potential application of PAdV vectors against major swine pathogens, including CSFV, PRRSV, PEDV/TGEV, SIV, and ASFV. It also illustrates key immunological outcomes, including innate immune activation, neutralizing antibodies, mucosal IgA, CD4+ and CD8+ T-cell responses, and IFN-γ production. Major translational issues, including anti-vector immunity, genetic stability, biosafety, manufacturing, and DIVA compatibility, are integrated with future priorities such as modular vaccine design, mucosal delivery, multivalent platforms, and scalable production.

This review synthesizes current knowledge on PAdV-based viral vectors for swine vaccine development, focusing on PAdV biology, recombinant vector engineering, PAdV-based vaccine studies, adenovirus-vectored vaccines for major swine diseases, immunological outcomes, advantages, limitations, biosafety considerations, comparative positioning, and future perspectives. By integrating direct PAdV evidence with broader adenovirus-vectored vaccine studies in pigs, this review provides a framework for evaluating PAdV as an emerging next-generation vaccine platform for the swine industry. The conceptual framework of PAdV-based viral vectors for swine vaccines is summarized in Figure 1, linking PAdV biology, vector engineering, vaccine applications, immune outcomes, translational considerations, and future development priorities.

Compared with previous reviews on porcine adenovirus biology, early PAdV vector development, and nonhuman adenoviral platforms, this review provides an updated scoping synthesis focused specifically on PAdV-based vectors for swine vaccines. Its added value lies in integrating foundational PAdV-3 studies with recent PAdV-5 isolation and reverse-genetics advances, separating direct PAdV vaccine evidence from comparative adenovirus-vectored swine vaccine evidence, and translating this evidence into disease-specific design lessons and evidence-based research priorities for target-animal validation, biosafety, manufacturing, and regulatory development.

### 1.1. Review Scope and Synthesis Approach

This review was structured as a scoping synthesis to map available evidence on PAdV biology, vector engineering, experimental PAdV-based vaccination, and comparative adenovirus-vectored vaccine studies relevant to swine pathogens. Because direct PAdV vaccine studies remain limited and heterogeneous, the synthesis integrates direct PAdV evidence with broader adenoviral-vector studies in pigs to identify current knowledge, recurring immune outcomes, translational gaps, and future research priorities.

The review emphasizes qualitative evidence mapping rather than meta-analysis. Key information was charted according to vector backbone, antigen insert, target pathogen, route of administration, immune endpoints, protection outcomes, biosafety considerations, and translational relevance. This approach is appropriate for an early-stage platform where study designs, endpoints, challenge models, and vector systems are not sufficiently comparable for quantitative pooling.

### 1.2. Review Scope and Literature Search Approach

A literature search was conducted to identify studies relevant to PAdV and adenovirus-based vaccine vectors for swine diseases. The databases searched included PubMed, Scopus, Web of Science, and Google Scholar. The search strategy combined disease-, vector-, and application-related terms using Boolean operators. Core search strings included: (“porcine adenovirus” OR “PAdV” OR “porcine mastadenovirus”) AND (“vector” OR “viral vector” OR “vaccine” OR “recombinant”); (“porcine adenovirus 3” OR “PAdV-3”) AND (“expression vector” OR “E1 deleted” OR “E3” OR “recombinant”); (“porcine adenovirus 5” OR “PAdV-5”) AND (“reverse genetics” OR “vaccine vector” OR “recombinant virus”); and (“adenovirus vector” OR “adenoviral vector”) AND (“swine” OR “pig” OR “porcine”) AND (“CSFV” OR “PRRSV” OR “PEDV” OR “TGEV” OR “SIV” OR “ASFV”).

Studies were selected if they reported PAdV biology, recombinant PAdV construction, PAdV-based antigen expression, experimental PAdV vaccination, or adenovirus-vectored vaccine studies relevant to major swine pathogens. Comparative studies using non-PAdV adenoviral platforms were included when they provided information relevant to antigen design, immune outcomes, delivery routes, safety, or translational development in pigs. Articles not related to adenoviruses, swine pathogens, vaccine vectors, or veterinary vaccine development were excluded.

### 1.3. Screening, Evidence Categorization, and Included Study Groups

Retrieved records were screened first by title and abstract, followed by full-text assessment when the study was potentially relevant to PAdV biology, recombinant PAdV construction, PAdV-based antigen expression, adenovirus-vectored swine vaccines, immune responses, biosafety, manufacturing, or regulatory translation. Studies were included if they provided evidence relevant to one or more of the following categories: (i) PAdV biology and genome/vector-engineering studies; (ii) direct PAdV-based vaccine studies; (iii) comparative adenovirus-vectored vaccine studies in pigs; (iv) studies addressing immunological endpoints relevant to swine vaccination; and (v) studies addressing biosafety, manufacturing, regulatory, or translational considerations (Table 1).

Review articles were used primarily for background, historical context, and synthesis, whereas primary research articles were prioritized when describing specific experimental findings. Because the available studies differed substantially in vector backbone, antigen insert, route of administration, animal model, immune endpoint, dose, challenge design, and outcome reporting, the synthesis was qualitative rather than quantitative. No quantitative effect-size synthesis was attempted because the included studies were not sufficiently comparable in design, endpoints, or outcome reporting.

## 2. Biology of PAdVs Relevant to Vector Development

PAdVs are non-enveloped, double-stranded DNA viruses in the family Adenoviridae. Like other mastadenoviruses, they possess an icosahedral capsid and a linear genome organized into early and late transcriptional regions. Early genes regulate viral replication and host-cell interactions, whereas late genes encode structural proteins required for virion assembly. These biological properties, together with genome stability, genetic manipulability, and capacity for heterologous gene expression, make adenoviruses attractive vaccine delivery platforms [5,7,8]. In swine vaccinology, PAdVs are particularly relevant because they are naturally associated with pigs and may provide a species-relevant vector backbone.

Among known PAdV, PAdV-3 and PAdV-5 have received the greatest attention for vector development. PAdV-3 is the best-characterized PAdV and was the first PAdV platform extensively investigated as an expression vector. Its complete genome sequence and transcriptional map revealed an approximately 34.1-kb genome with an adenovirus-like organization and distinctive features, including high G+C content [13]. Subsequent studies showed that PAdV-3 could be engineered to express foreign genes and developed as a recombinant vaccine vector [14,15].

Several genomic regions are important for PAdV engineering. The E1 region is essential for replication and can be deleted to generate replication-defective vectors propagated in complementing cells. This strategy was successfully adapted to PAdV-3, demonstrating the feasibility of replication-defective recombinant PAdV systems [15]. The E3 region is another potential insertion site, although PAdV biology suggests that presumed non-essential regions must be evaluated carefully before deletion or replacement [34,35].

Capsid proteins also influence vector performance. Hexon is important for classification, whereas fiber mediates host-cell attachment, receptor usage, tissue tropism, host range, and anti-vector immunity. Structural analysis of porcine adenovirus type 4 (PAdV-4) fiber domains provided insight into capsid architecture and receptor-related properties [36]. PAdV-3 also differs from HAdV-5 in cell entry because its internalization is independent of Coxsackievirus and adenovirus receptor (CAR) and αvβ3/αvβ5 integrins, which may affect tropism, transduction, and evasion of anti-HAdV immunity [37].

PAdV-5 has recently attracted renewed interest. Molecular characterization showed that PAdV-5 can be detected in diarrheic pigs, and conserved genes such as DNA polymerase and hexon are useful for strain comparison [17]. More recently, PAdV-5 isolation and reverse genetic manipulation supported its potential as an emerging veterinary vector platform [18]. Overall, PAdV vectors offer species relevance, foreign gene insertion capacity, and potential for replication-competent or replication-defective design. However, further work is needed on serotype diversity, receptor usage, tissue tropism, anti-vector immunity, genetic stability, safety, and scalable producer cell systems before PAdV can become a practical swine vaccine platform [5,34].

## 3. Engineering Strategies for PAdV-Based Vectors

**Figure 2:**
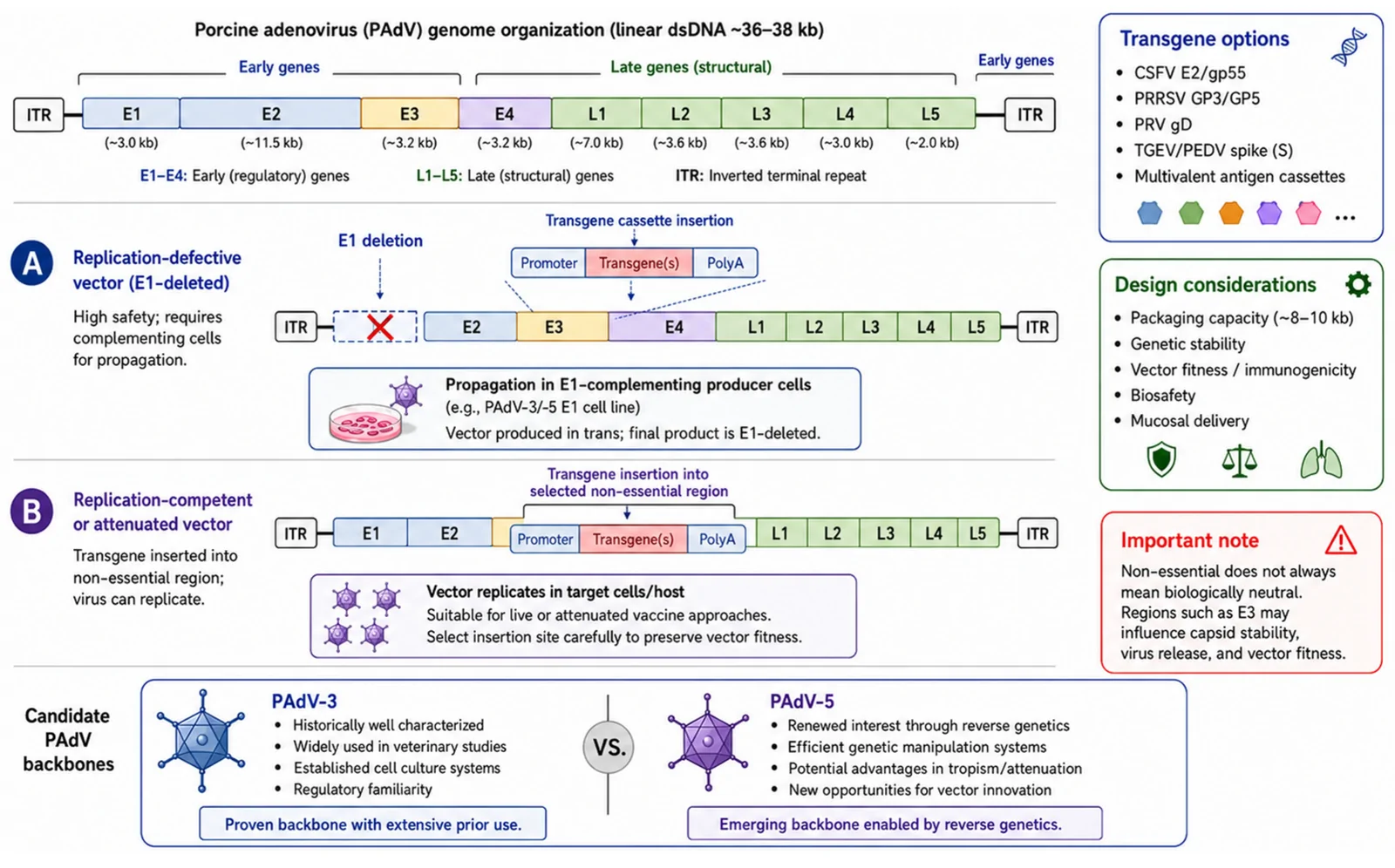
Engineering strategies for PAdV vaccine vectors. This figure illustrates major engineering strategies for porcine adenovirus vaccine-vector development. The schematic shows the general PAdV genome organization, including inverted terminal repeats, early regulatory regions, and late structural genes. Two principal vector-design approaches are shown: replication-defective vectors, typically generated by deletion of essential regions such as E1 and propagated in complementing producer cells; and replication-competent or rationally attenuated vectors, in which transgene cassettes are inserted into selected genomic regions while preserving vector fitness. Candidate antigen inserts include CSFV E2/gp55, PRRSV GP3/GP5, PRV-gD, TGEV/PEDV spike protein, and multivalent antigen cassettes. The figure also highlights major design constraints, including packaging capacity, genetic stability, vector fitness, biosafety, and mucosal delivery potential. The comparison between PAdV-3 and PAdV-5 emphasizes that PAdV-3 is historically well characterized, whereas PAdV-5 has renewed relevance because of recent advances in isolation, characterization, and reverse genetics. Genome organization and approximate genome size are based on published PAdV-3 and PAdV-5 genomic studies, whereas insert-capacity considerations are inferred from reported recombinant PAdV construction studies and broader adenoviral-vector engineering principles. Exact packaging limits and tolerated insert sizes may differ among PAdV serotypes, deletion backgrounds, insertion sites, and vector backbones and therefore require empirical validation.

The development of PAdV-based vectors depends on the ability to manipulate the viral genome while preserving essential functions required for virion rescue, propagation, transgene expression, and immunogenicity. Engineering strategies for PAdV vectors have generally followed principles established for other adenoviral platforms, particularly the use of non-essential or conditionally essential genomic regions for deletion, insertion, attenuation, or replacement with heterologous antigen expression cassettes. However, PAdV vectors also require species-specific optimization because PAdVs differ from HAdV in genome sequence, transcriptional organization, receptor usage, host-cell interaction, and growth characteristics [13,34,37]. Therefore, PAdV vector engineering must balance antigen expression, genetic stability, replication control, production efficiency, and safety. The major engineering strategies for PAdV vaccine-vector development are illustrated in Figure 2.

### 3.1. Selection of PAdV Serotype and Vector Backbone

The first step in PAdV vector design is the selection of a suitable viral backbone. PAdV-3 has historically been the most extensively investigated PAdV vector platform. The complete genome sequence and transcription map of PAdV-3 provided the basis for identifying essential and non-essential regions suitable for genetic manipulation [13]. Subsequent work demonstrated that PAdV-3 could be engineered as an expression vector and could support insertion and expression of foreign genes [14]. These studies established PAdV-3 as the prototype porcine adenoviral vector and provided a foundation for later development of replication-defective and helper-dependent systems.

PAdV-5 has recently gained interest as an alternative vector backbone. Earlier work on PAdV-5 demonstrated that its E3 region could be sequenced and deleted, supporting the feasibility of genetic manipulation [38]. Recombinant PAdV-5 expressing the TGEV spike gene was subsequently constructed and characterized, providing proof-of-concept evidence that PAdV-5 could carry and express a major swine coronavirus antigen [16]. More recently, the isolation of a PAdV-5 strain and establishment of reverse genetic manipulation renewed interest in this serotype as an emerging platform for veterinary vaccine development [18]. These findings suggest that PAdV-3 and PAdV-5 may represent complementary vector backbones, although direct comparisons of their transgene capacity, immunogenicity, seroprevalence, and production efficiency remain limited.

The main contribution of recent PAdV-5 work is not simply the addition of another serotype, but the possibility of re-evaluating PAdV vectors using modern reverse genetics. This enables more precise definition of essential and non-essential genomic regions, rational insertion-site testing, recombinant virus rescue, and direct comparison with the historically better characterized PAdV-3 backbone. Such studies could determine whether PAdV-5 offers practical advantages in antigen capacity, growth kinetics, genetic stability, mucosal tropism, or anti-vector immunity.

### 3.2. Deletion of the E1 Region and Generation of Replication-Defective Vectors

The E1 region is one of the most important targets for engineering adenoviral vectors. In adenoviruses, E1 genes regulate early viral transcription and are essential for efficient viral replication. Deletion of E1 can generate replication-defective vectors that are unable to replicate autonomously in normal host cells but can be propagated in complementing cell lines that provide E1 functions in trans. This strategy improves vector safety and is widely used in HAdV-based vaccine platforms. Similar principles have been applied to PAdV-3 vector development.

A major advance in PAdV engineering was the construction of recombinant E1-deleted PAdV-3 vectors. Zakhartchouk et al. [15] developed E1-deleted PAdV-3 expression vectors and established a complementing cell system that allowed rescue and propagation of replication-defective recombinant PAdV. In that study, fetal porcine retina-derived cells expressing PAdV-3 E1B large protein were used to support the rescue of recombinant viruses with complete E1 deletion. This work demonstrated that replication-defective PAdV vectors could be generated and propagated using appropriate helper cell systems, thereby extending the adenoviral vector design concept to PAdV.

Replication-defective PAdV vectors are attractive for vaccine development because they reduce the risk of uncontrolled replication, environmental spread, and reversion to virulence. They may be especially suitable for vaccines intended for broad field use, repeated administration, or combination with other immunization strategies. However, E1-deleted PAdV vectors also require efficient complementing cell lines for production, and this requirement remains one of the major technical barriers to translational development. Producer cells must support high-titer vector amplification, maintain genetic stability, and meet regulatory expectations for veterinary biological products.

### 3.3. Modification of the E3 Region and Transgene Insertion

The E3 region is another important target for PAdV vector engineering. In many adenoviruses, E3 genes are involved in modulation of host immune responses but are not essential for viral replication in cell culture. Therefore, E3 deletion can provide space for insertion of foreign gene expression cassettes without completely abolishing viral replication. This strategy has been used in several adenoviral vector systems and is particularly useful when replication-competent vectors are desired.

In PAdV-5, the E3 region has been sequenced and experimentally deleted, supporting its use as a candidate insertion site [38]. A recombinant PAdV-5 vector expressing the TGEV spike gene was constructed using this engineering principle, demonstrating that the PAdV genome could accommodate and express a large coronavirus antigen [16]. Similarly, earlier PAdV-based vaccine studies used recombinant vectors expressing antigens from CSFV and PRV, indicating that PAdV vectors can deliver heterologous genes from diverse swine pathogens [20,21,22].

Although E3 deletion can increase insertion capacity, the biological role of E3 genes may differ among adenovirus species. Recent work on PAdV-3 has suggested that E3 may encode a structural protein important for capsid stability and production of infectious progeny virions [39]. This finding is important because it indicates that assumptions derived from HAdV may not always apply directly to PAdV. Therefore, deletion or replacement of the E3 region in PAdV should be evaluated carefully for effects on viral growth, particle stability, infectivity, transgene expression, and vaccine yield.

### 3.4. Replication-Competent Versus Replication-Defective PAdV Vectors

PAdV vectors can theoretically be developed as either replication-competent or replication-defective platforms. Replication-competent vectors may provide prolonged antigen expression and stronger mucosal immune stimulation, particularly when administered orally or intranasally. Such vectors may be useful for enteric or respiratory swine diseases where local immunity is important. Early recombinant PAdV vaccination studies, including those targeting CSFV and TGEV, provided evidence that PAdV-based vectors can be administered by different routes and induce measurable immune responses in pigs [16,20,21,22].

However, replication-competent vectors raise safety concerns, including shedding, transmission to contact animals, environmental persistence, and recombination with circulating field strains. These concerns are particularly relevant for livestock vaccines used in large populations. In contrast, replication-defective vectors may offer a more controllable platform because productive replication is restricted in normal host cells, although biodistribution, residual particle release, recombination risk, and production-related contaminants still require evaluation. Their main disadvantages are the need for complementing production cell lines and the possibility of reduced antigen expression compared with replication-competent vectors. The optimal design depends on the target pathogen, immunization route, desired immune profile, animal age, maternal antibody status, and regulatory requirements. At present, direct comparative studies between replication-competent and replication-defective PAdV vectors in pigs remain limited, representing an important research gap.

### 3.5. Transgene Design and Expression Cassette Optimization

Efficient antigen expression is central to the performance of recombinant PAdV vaccines. Transgene design must consider antigen size, codon usage, signal peptides, membrane anchoring, secretion, folding, post-translational modification, and compatibility with the adenoviral genome. For swine pathogens, antigens commonly considered for adenoviral expression include envelope glycoproteins, surface proteins, capsid proteins, and conserved T-cell antigens. Examples include the E2 or gp55 antigen of CSFV, gD of PRV, and spike protein of TGEV [16,20,21,22].

Expression cassettes usually require a strong promoter, polyadenylation signal, and appropriate cloning orientation. Cytomegalovirus immediate-early promoters have been widely used in adenoviral vectors because they support strong transgene expression in many mammalian cell types. However, promoter selection may influence tissue-specific expression, duration of antigen production, vector fitness, and immune outcome. Future PAdV vector designs may benefit from systematic comparison of promoters, including constitutive, tissue-selective, and immune-cell-active promoters. In addition, antigen expression may be enhanced by codon optimization, secretion signals, multimerization domains, or fusion to immunostimulatory molecules, although these strategies remain underexplored in PAdV-based systems.

### 3.6. Recombinant Virus Rescue and Reverse Genetics

Traditional PAdV vector construction has relied on homologous recombination, plasmid-based cloning, or transfection of viral genomic DNA into permissive or complementing cells. These methods can be technically demanding, particularly for large adenoviral genomes that may be unstable in bacterial systems. The development of simplified recombinant adenovirus generation methods has therefore been important for accelerating vector design. Zhang et al. [40] described a rapid method for generating PAdV vectors, supporting more efficient manipulation of PAdV genomes and facilitating experimental vector construction.

Reverse genetics is especially valuable because it allows precise genomic manipulation, rescue of recombinant viruses from cloned viral genomes, and systematic identification of essential and non-essential regions. The recent construction of a recombinant PAdV-5 platform using reverse genetics represents an important advance for the field [18]. Such systems can support rational design of deletion mutants, insertion of vaccine antigens, reporter gene expression, attenuation studies, and comparative testing of vector variants. As reverse genetic systems become more efficient, they are likely to accelerate the development of next-generation PAdV vectors for swine vaccines.

### 3.7. Genetic Stability, Packaging Capacity, and Vector Fitness

Genetic stability is a critical requirement for PAdV-based viral vector vaccines. Recombinant PAdV vectors must retain inserted antigen genes during serial passage, production, storage, and in vivo administration. Instability may cause transgene deletion, reduced antigen expression, loss of efficacy, or accumulation of defective particles. Packaging capacity is also an important constraint because genome size and insertion site can influence virion packaging, replication efficiency, and vector stability. HAdV-5 studies showed that packaging capacity and genome size are closely linked to genetic stability, suggesting that oversized or poorly balanced recombinant genomes may become unstable during propagation [41]. Similar principles likely apply to PAdV, although the precise packaging limits of different PAdV serotypes and deletion backgrounds remain incompletely defined. Earlier PAdV reviews suggested that adenoviral genome architecture constrains insert size and that deletion of non-essential regions may be needed to accommodate larger transgenes [34,35].

Vector fitness must also be considered because large inserts or disruption of important genomic regions may reduce replication, particle production, infectivity, or capsid stability. The finding that the PAdV-3 E3 region encodes a 13.7 K structural protein required for capsid stability and infectious progeny production shows that presumed deletion sites must be empirically validated [39]. Recent PAdV-5 reverse genetics further supports defining essential and non-essential regions before vector use [18]. Future PAdV vaccines should therefore undergo serial-passage testing, restriction analysis or full-genome sequencing, antigen-expression assays, infectious-titer measurement, and assessment of defective or replication-competent revertant particles.

### 3.8. Producer Cell Systems and Manufacturing Considerations

Efficient production is essential for translating PAdV vectors into practical vaccines. Replication-competent vectors can be propagated in permissive porcine cell lines, whereas replication-defective vectors require complementing cell lines that provide deleted viral functions. Zakhartchouk et al. [15] demonstrated the feasibility of complementing E1-deleted PAdV-3 using engineered cells, but broader development of stable, high-yield, regulatory-acceptable producer cells remain limited. This issue is a major difference between PAdV and HAdV platforms, which benefit from decades of optimization in cell lines such as HEK293 or PER.C6-like systems.

For veterinary vaccine production, cell substrates must support consistent high-titer growth, be scalable, and comply with safety standards. Additional concerns include adventitious agents, genetic stability of complementing functions, risk of replication-competent revertants, serum-free culture adaptation, purification processes, and batch-to-batch consistency. These manufacturing considerations are not only technical issues but also determine whether PAdV vectors can progress from experimental constructs to commercial vaccine candidates.

### 3.9. Engineering Priorities for Platform Development

Future PAdV engineering should prioritize experimentally validated insertion sites, stable E1- or E1/E3-modified backbones, efficient reverse-genetics systems, and producer cells that support high-titer and genetically stable vector rescue. These priorities are not simply technical refinements; they determine whether PAdV can move from antigen-expression constructs to reproducible vaccine candidates suitable for target-animal testing and manufacturing evaluation [14,15,18,34,39].

At present, the engineering evidence supports feasibility rather than platform maturity. Therefore, future PAdV constructs should be assessed using serial-passage stability, whole-genome sequencing, antigen-expression assays, infectious-titer measurement, and testing for replication-competent or defective particles before efficacy claims are advanced.

## 4. PAdV-Based Vaccine Studies

PAdV-based vaccine studies remain limited in number, but they provide important proof-of-concept evidence that PAdV vectors can express heterologous antigens and induce protective or partially protective immune responses in pigs. Most experimental studies were performed during the early phase of PAdV vector development and focused on economically important swine pathogens, including CSFV, PRV, and TGEV. These studies demonstrated the feasibility of using recombinant PAdV vectors for antigen delivery through parenteral and mucosal routes. However, differences in vector backbone, antigen insert, immunization regimen, route of administration, animal age, immune readout, and challenge design make direct comparison difficult. Therefore, the available evidence should be interpreted as an early developmental foundation rather than a mature vaccine platform. Representative direct evidence for PAdV-based vaccine development is summarized in Table 2.

### 4.1. Classical Swine Fever Virus (CSFV)

CSFV is the most extensively studied target pathogen for PAdV-based vaccination. CSFV is a highly contagious pestivirus of domestic pigs and wild boar that causes severe systemic disease and major economic losses. Therefore, vaccine development must consider both protective immunity and disease-control policies. The major envelope glycoprotein E2, historically known as gp55, is a key protective antigen widely used in CSFV vaccine design.

One of the earliest PAdV-based vaccine candidates was a recombinant PAdV expressing CSFV gp55/E2. Hammond et al. [19] showed that a single dose of recombinant PAdV-gp55/E2 protected pigs against CSFV challenge, providing proof-of-concept evidence that PAdV can deliver a protective antigen and function as an in vivo vaccine vector in pigs. Subsequent studies evaluated oral and subcutaneous vaccination in commercial pigs. Both routes induced immune responses, although magnitude and consistency differed by route [23]. In-contact challenge studies further showed that two subcutaneous doses increased CSFV-neutralizing antibody responses compared with a single dose, but protection varied according to route and regimen [21].

A heterologous strategy using plasmid DNA priming followed by recombinant PAdV boosting also protected pigs against CSF, supporting the use of PAdV in sequential vaccination regimens [22]. Overall, CSFV studies provide the strongest evidence for PAdV-based vaccination, showing that recombinant PAdV can express a protective glycoprotein, induce neutralizing antibodies, and contribute to protection. However, these studies used small animal numbers and early vector designs and did not fully address long-term immunity, field efficacy, pre-existing PAdV immunity, manufacturing scalability, or DIVA compatibility.

### 4.2. Pseudorabies Virus (PRV)

PRV, also known as suid herpesvirus 1, is another important swine pathogen that was investigated using recombinant PAdV-based vaccination. gD is a major immunogenic envelope protein of PRV and is involved in virus entry and induction of neutralizing antibodies. Because gD has been used in several herpesvirus vaccine platforms, it represented a logical antigen for testing recombinant PAdV delivery.

Hammond et al. [20] evaluated vaccination of pigs with a recombinant PAdV expressing the PRV-gD gene. Five-week-old commercial pigs were vaccinated with either a single dose or two doses of the recombinant PAdV-gD vector. The study demonstrated that PAdV could express a herpesvirus antigen and stimulate antigen-specific immune responses in pigs. Although this work was smaller in scope than the CSFV studies, it was important because it expanded the application of PAdV beyond pestivirus antigens and showed that recombinant PAdV could deliver glycoprotein antigens from a DNA virus.

The recombinant PAdV-gD study in the pseudorabies virus/Aujeszky’s disease model also helped establish the broader antigen-delivery capacity of PAdV vectors. Expression of gD suggested that PAdV could be used for envelope glycoproteins that require appropriate folding and antigenic presentation. This is relevant for future PAdV-based vaccine development because many important swine pathogens require induction of neutralizing antibodies against surface glycoproteins. However, the study provided limited information on protective efficacy, mucosal immunity, duration of immunity, or comparison with existing AD vaccines. Therefore, recombinant PAdV-gD should be viewed primarily as proof of antigen expression and immunogenicity rather than a fully validated vaccine candidate.

### 4.3. Transmissible Gastroenteritis Virus (TGEV)

TGEV is an enteric coronavirus of pigs, and its spike protein is the major target for neutralizing antibody responses. Because protection against enteric coronaviruses often requires mucosal immunity, TGEV provided a relevant model for evaluating whether PAdV vectors could be used for enteric vaccine applications.

Tuboly and Nagy [16] constructed and characterized a recombinant PAdV serotype 5 expressing the TGEV spike gene. This study followed earlier work on sequencing and deleting the PAdV-5 E3 region, which provided a genomic basis for insertion of foreign genes [38]. The recombinant PAdV-5 expressing TGEV-S protein demonstrated that PAdV-5 could be engineered to carry a large coronavirus antigen. This was an important technical achievement because coronavirus spike proteins are large, structurally complex glycoproteins, and their expression can be challenging in viral vector systems.

The TGEV spike study provided evidence that PAdV-5 could serve as a recombinant vector for enteric swine coronavirus antigens. However, the available data were primarily focused on construction and characterization rather than comprehensive vaccine efficacy in pigs. Therefore, while this study supports the feasibility of PAdV-5 vector engineering, further work would be required to determine whether PAdV-5-based coronavirus vaccines can induce strong mucosal immunity, lactogenic immunity, neutralizing antibodies, and protection against enteric challenge. These questions are particularly relevant for future application of PAdV vectors against porcine epidemic diarrhea virus (PEDV) and other swine coronaviruses.

### 4.4. Lessons and Current Status of PAdV-Based Vaccine Evidence

Taken together, early PAdV-based studies provide direct proof-of-concept evidence that PAdV vectors can express heterologous antigens from diverse virus families and induce immune responses in pigs. Recombinant PAdV-3 expressing CSFV gp55/E2 induced antigen expression, neutralizing antibodies, and protection, while PAdV-based delivery of PRV-gD and PAdV-5 expression of TGEV-S expanded the antigenic scope of the platform [16,19,20,21,22,23]. These findings support technical feasibility, but they do not yet establish PAdV as a mature commercial vaccine platform.

The major limitation is the age and scale of the evidence base. Most studies were performed more than two decades ago using early vector designs, small animal numbers, and limited immunological tools [34]. They did not comprehensively evaluate cellular immunity, mucosal IgA, innate responses, duration of immunity, maternal antibody interference, anti-vector immunity, shedding, genetic stability, or scalable production. Recent PAdV-5 isolation and reverse genetics have renewed interest in the platform, but modern target-animal efficacy, biosafety, and manufacturability remain to be demonstrated [17,18].

Thus, PAdV should be interpreted as a biologically plausible and technically feasible swine-associated adenoviral vector, but current evidence remains proof-of-concept. Future studies must move beyond antigen-expression readouts and use standardized immune endpoints, target-animal challenge models, biosafety testing, and production-feasibility assessment [5,8,9,10,34]. Accordingly, the following section considers broader adenovirus-vectored vaccine evidence only as comparative guidance for future PAdV development, not as direct proof of PAdV efficacy.

## 5. Comparative Adenovirus-Vectored Vaccine Evidence for Major Swine Diseases

**Figure 3:**
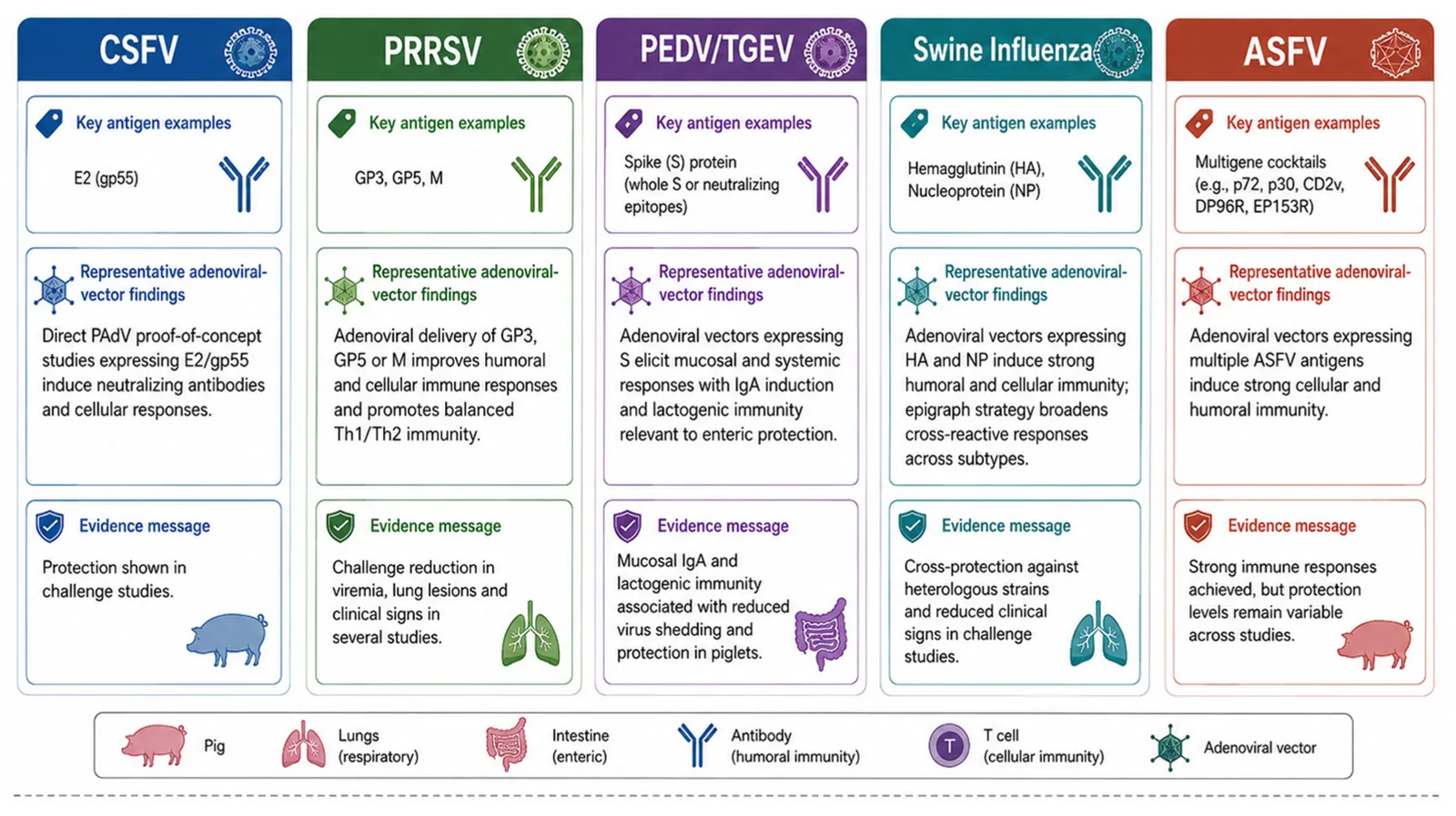
Vaccine applications of PAdV and adenoviral vectors for major swine diseases. This figure summarizes direct and comparative evidence supporting the use of PAdV and other adenoviral vectors for major swine viral diseases. The disease panels include CSFV, PRRSV, PEDV/TGEV, SIV, and ASFV. For each pathogen, representative antigen targets, adenoviral-vector findings, and key evidence messages are shown. Direct PAdV evidence is strongest for CSFV, where recombinant PAdV expressing E2/gp55 induced neutralizing antibodies and protection in pigs. Comparative adenoviral-vector studies for PRRSV, PEDV/TGEV, SIV, and ASFV demonstrate the broader potential of adenoviral platforms to deliver structural antigens, conserved antigens, mucosal targets, or multigene antigen cocktails. The figure highlights that vaccine performance depends on antigen selection, immune endpoint, delivery route, and vector-regimen design.

Although direct studies of PAdV-based vaccines remain limited, adenovirus-vectored vaccines have been extensively investigated for several major swine diseases. These studies provide important comparative evidence for evaluating the potential of PAdV as a future swine vaccine platform. Most non-PAdV adenoviral vaccine studies in pigs have used HAdV, CAdV, or other recombinant adenoviral backbones to deliver viral antigens. The results of these studies are useful for understanding antigen selection, immune profiles, the route of administration, prime-boost strategies, protection outcomes, and translational barriers that may also apply to PAdV-based vectors. The potential applications of PAdV and other adenoviral vectors against major swine viral diseases are summarized in Figure 3. Comparative evidence from adenovirus-vectored vaccine studies targeting major swine diseases is summarized in Table 3. To avoid repetition, Section 5 focuses primarily on comparative adenovirus-vectored vaccine evidence and disease-specific design lessons, whereas direct PAdV-based vaccine studies are discussed in Section 4. Direct PAdV examples are mentioned only where necessary to link comparative evidence to future PAdV development. Because direct PAdV data are available only for selected pathogens, Table 3 separates direct PAdV evidence from comparative adenoviral-vector evidence derived from other platforms. Comparative evidence is included to identify design principles but should not be interpreted as proof of PAdV efficacy for diseases where direct PAdV studies have not yet been performed.

### 5.1. African Swine Fever Virus (ASFV)

ASFV is one of the most difficult targets for swine vaccine development because of its large genome, complex antigenic structure, immune evasion, and high mortality in domestic pigs and wild boars. Adenovirus-vectored ASFV vaccines are attractive because they can express multiple antigens and induce both antibody and T-cell responses. Early adenovirus-vectored ASFV antigen cocktails induced robust antigen-specific antibody and cellular immune responses in pigs, but strong immunogenicity did not consistently result in complete protection, indicating that antigen selection and immune correlates remain major challenges [26,27].

Subsequent studies showed that protection varies according to antigen combination, vector regimen, challenge strain, and delivery strategy. Adenovirus-vectored ASFV antigen cocktails were immunogenic but failed to protect pigs against intranasal challenge with the virulent Georgia 2007/1 isolate [32]. In contrast, selected virally vectored ASFV antigen pools protected pigs against fatal disease under specific conditions, and more recent antigen combinations or adenovirus-vectored regimens also conferred protection in swine [31,48,49].

These studies provide important lessons for future PAdV development. PAdV vectors could be useful for expressing multiple ASFV antigens, but protection will require careful antigen selection, evaluation of IFN-γ-producing and cytotoxic T-cell responses, and optimization of heterologous prime-boost or repeated immunization regimens while considering anti-vector immunity.

### 5.2. Porcine Epidemic Diarrhea Virus (PEDV) and Other Swine Coronaviruses

PEDV is an enteric coronavirus causing severe diarrhea, dehydration, and high mortality in neonatal piglets, resulting in major economic losses. Protection depends strongly on mucosal and lactogenic immunity, especially secretory IgA and neutralizing antibodies transferred from immunized sows through colostrum and milk. Therefore, PEDV is a relevant model for evaluating adenovirus-vectored vaccines that induce intestinal and maternal immune responses [1,3,50,51]. Recombinant adenovirus expressing PEDV spike induced PEDV-specific immune responses in pigs, supporting adenoviral delivery of coronavirus spike antigens [3,28].

Mucosal adenovirus-vectored PEDV designs have also been explored. A recombinant adenovirus expressing the heat-labile enterotoxin B subunit and the PEDV core neutralizing epitope was designed to enhance mucosal immunogenicity, consistent with the need for intestinal immunity rather than only systemic immunity against swine enteric coronaviruses [50,51,52,53].

These findings are relevant to PAdV development because PAdV-5 expressing TGEV spike showed that PAdV can carry a large coronavirus spike antigen [16]. Future PAdV-based coronavirus vaccines should optimize mucosal delivery, local IgA, lactogenic immunity, spike expression, and genetic stability [1,3,34].

### 5.3. Porcine Reproductive and Respiratory Syndrome Virus (PRRSV)

PRRSV is one of the most economically important swine pathogens worldwide. Vaccine development is difficult because of viral genetic diversity, immune evasion, delayed neutralizing antibody responses, and incomplete cross-protection. Adenovirus-vectored PRRSV vaccines have therefore been explored to improve antigen-specific immunity, especially against structural proteins such as GP3, GP5, and M.

Cao et al. [25] showed that recombinant adenovirus expressing CD40 ligand together with PRRSV GP3 and GP5 enhanced immune responses, indicating that adenoviral vectors can deliver both antigens and immunostimulatory molecules. CAdV vectors expressing PRRSV GP5 and M proteins, or GP5 alone, also induced antigen-specific immune responses in pigs, demonstrating that non-HAdV vectors can be applied to PRRSV antigen delivery [42,43]. More recently, recombinant adenoviruses expressing GP3, GP5, or both antigens from an NADC34-like PRRSV strain induced humoral and cellular responses and reduced viral load after challenge, supporting updated antigen selection for emerging lineages [30].

For PAdV platforms, PRRSV studies highlight the need for rational antigen design, cellular immune endpoints, multivalent antigen expression, molecular adjuvants, and prime-boost strategies. Future PAdV vaccines could express GP5, M, GP3, conserved T-cell antigens, or immune modulators, but vector selection alone will not overcome PRRSV antigenic diversity without optimized antigen design.

### 5.4. Swine Influenza Virus (SIV)

SIV is an important respiratory pathogen of pigs with zoonotic significance, and conventional vaccines often provide limited cross-protection against antigenically diverse strains. Adenoviral vectors have therefore been explored for delivering influenza antigens, including hemagglutinin, nucleoprotein, and computationally designed immunogens. Early recombinant HAdV-5 vaccines expressing SI hemagglutinin and nucleoprotein protected weaned pigs and showed that adenoviral vectors could overcome some limitations of conventional vaccination [24,45]. Additional work indicated that adenoviral-vectored influenza vaccination may help overcome interference from maternally derived antibodies in young pigs [46].

Recent studies have focused on epitope-optimized influenza immunogens. An adenoviral-vectored epigraph vaccine induced robust, durable, and protective immunity against H3 influenza A virus in swine, while a later study showed reduced clinical disease, viral shedding, and lung lesions after experimental challenge [44,47]. These findings show that adenoviral vectors can be combined with computational antigen design to improve breadth and durability of protection.

For PAdV development, SI studies suggest that adenoviral vectors can express both structural and conserved antigens, may be useful in young pigs with maternal antibodies, and could benefit from respiratory mucosal delivery. Modern epigraph or consensus immunogens could be integrated into PAdV backbones for next-generation SI vaccines.

### 5.5. Pseudorabies Virus (PRV) and Other Herpesvirus Models

PRV has been used as a useful herpesvirus model for adenovirus-vectored vaccination in pigs. Because direct PAdV-based delivery of PRV-gD has already been discussed in Section 4.2, this section focuses on the broader comparative relevance of herpesvirus antigen delivery. Earlier adenovirus-vectored studies indicate that glycoprotein antigens can be delivered by adenoviral platforms and may support early-life vaccination or DIVA-oriented designs.

Because effective live attenuated and marker vaccines already exist for Aujeszky’s disease in many regions, the main value of PRV in this review is comparative rather than promotional. Future PAdV-based PRV vaccines would need to demonstrate clear added value over established approaches, such as improved safety, DIVA compatibility, mucosal delivery, early-life immunization, or compatibility with existing eradication programs [54].

### 5.6. Classical Swine Fever Virus (CSFV) and Pestivirus Models

Because direct PAdV-based CSFV vaccination studies are discussed in detail in Section 4.1, this section emphasizes their broader design implications. CSFV provides a useful pestivirus model showing that adenoviral delivery of a defined protective glycoprotein antigen can induce functional neutralizing antibodies and contribute to protection in pigs. This supports the general principle that PAdV vectors may be suitable for antigens with well-defined protective targets.

However, future PAdV-based CSFV vaccines would need to show added value over existing live attenuated and E2 subunit vaccines. Their potential commercial or field relevance will depend on DIVA compatibility, safety, production cost, stability, the route of administration, surveillance compatibility, and trade-related disease-control requirements [55,56,57,58,59].

### 5.7. Comparative Lessons from Adenovirus-Vectored Swine Vaccines

Across major swine diseases, adenovirus-vectored vaccine studies provide three cross-cutting lessons for PAdV development. First, adenoviral vectors can deliver diverse antigens and induce humoral, cellular, and sometimes mucosal responses in pigs, but vector immunogenicity alone is not a reliable surrogate for protection. This is particularly evident for ASFV and PRRSV, where strong immune responses do not consistently predict complete protection. Second, antigen selection and disease biology are more decisive than vector choice alone. Third, route and regimen must be matched to pathogen entry and immune correlates: enteric pathogens require intestinal and lactogenic immunity, respiratory pathogens require local mucosal and T-cell responses, and systemic pathogens may require neutralizing antibodies together with strong cellular immunity.

These comparative findings should therefore be used as design guidance rather than indirect proof of PAdV efficacy. For enteric pathogens, PAdV should be optimized for mucosal and lactogenic endpoints; for respiratory pathogens, intranasal or aerosol delivery should be evaluated for nasal IgA, lung T-cell responses, shedding reduction, and safety; for ASFV and CSFV, multivalent antigen design, cellular immunity, viremia reduction, and challenge protection are more relevant than antibody magnitude alone. The disease-specific evidence and its relevance to PAdV design are summarized in Table 3 and Figure 3.

Overall, broader adenovirus-vectored swine vaccine studies justify renewed PAdV research, but they also highlight the need for caution. Future PAdV studies should directly test whether a porcine adenoviral backbone provides measurable advantages over established adenoviral vectors when the same antigen, dose, route, and challenge model are used.

## 6. Immunological Outcomes Induced by Adenoviral Vectors in Pigs

**Figure 4:**
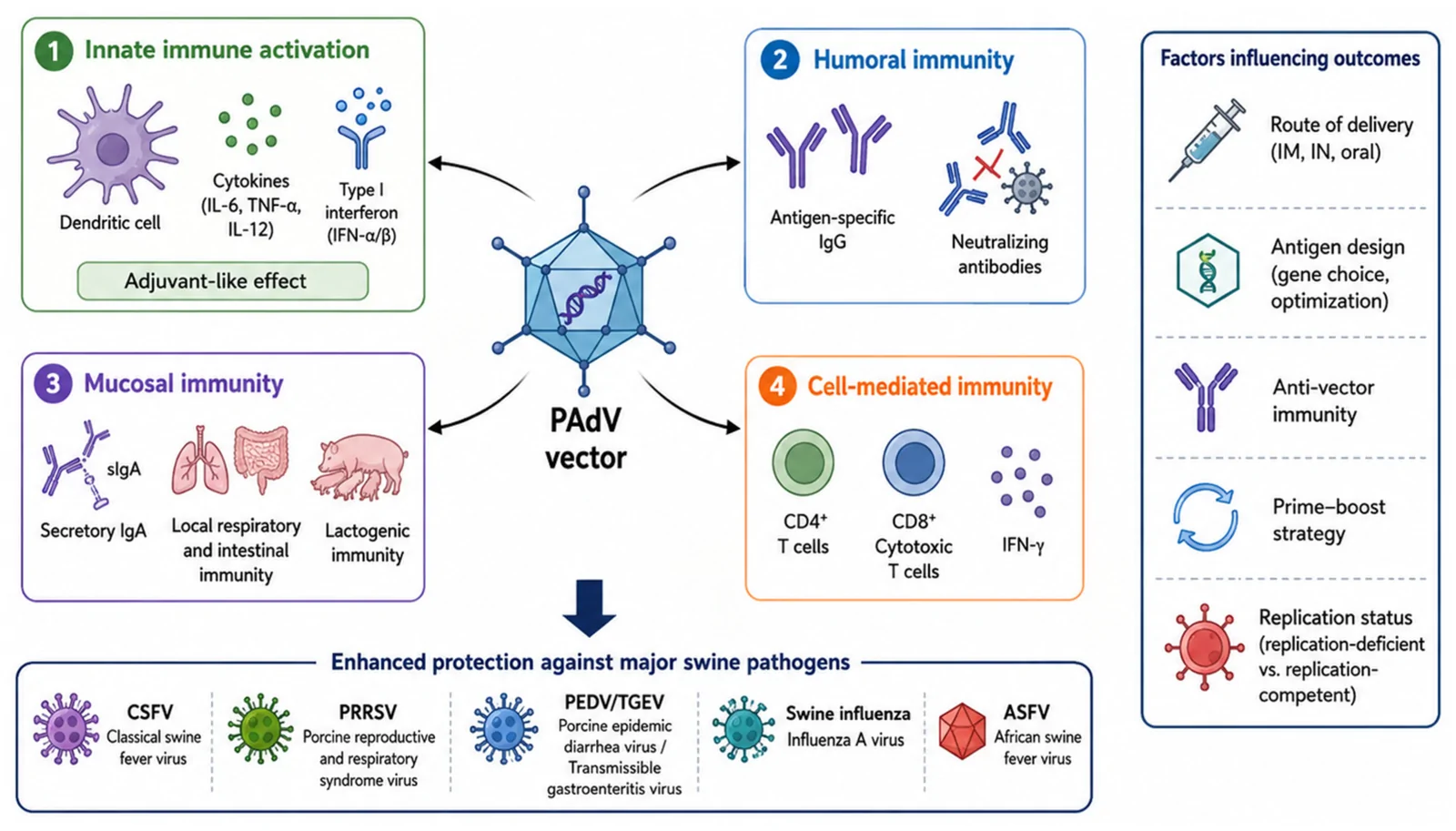
Immunological outcomes and evidence levels relevant to PAdV-based swine vaccine development. This figure summarizes the major immune responses induced or expected following adenoviral-vector vaccination in pigs. Adenoviral vectors can activate innate immune pathways through viral particles, capsid proteins, and vector DNA, leading to dendritic cell activation, cytokine production, type I interferon responses, and adjuvant-like effects. They can also induce humoral immunity, including antigen-specific IgG and neutralizing antibodies, which are particularly important for glycoprotein-based antigens such as CSFV E2, PEDV/TGEV spike, and SIV hemagglutinin. Mucosal immunity, including secretory IgA, respiratory and intestinal immune responses, and lactogenic immunity, is especially relevant for enteric and respiratory swine pathogens. Cell-mediated immunity, including CD4+ T-cell responses, CD8+ cytotoxic T-cell responses, and IFN-γ production, is important for complex viral infections such as ASFV, PRRSV, and swine influenza. The figure also illustrates key modifiers of vaccine outcome, including delivery route, antigen design, anti-vector immunity, prime–boost strategy, and replication status. Direct PAdV-mediated protective evidence is currently strongest for CSFV, with additional proof-of-concept antigen delivery evidence for PRV and TGEV. Immune outcomes for PEDV, PRRSV, SIV, and ASFV are derived mainly from comparative adenovirus-vectored vaccine studies or represent proposed endpoints for future PAdV evaluation. Therefore, this figure should be interpreted as an immunological framework rather than evidence that PAdV has already conferred protection against all listed swine diseases.

Adenoviral vectors are attractive vaccine platforms because they can induce multiple layers of immune responses, including innate immune activation, antigen-specific antibody production, mucosal immunity, and cellular immunity. In pigs, these immunological properties are particularly relevant because many important swine pathogens infect mucosal surfaces, evade host immunity, or require both humoral and cell-mediated responses for effective protection. Evidence from PAdV-based vaccine studies and broader adenovirus-vectored vaccine studies in pigs indicates that adenoviral vectors can stimulate antigen-specific immune responses, although the magnitude, quality, and protective relevance of these responses vary according to the vector backbone, antigen design, route of administration, vaccination regimen, animal age, and challenge model. The main immunological outcomes induced or expected after adenoviral-vector vaccination in pigs are summarized in Figure 4. These responses include innate immune activation, humoral immunity, mucosal immunity, and cell-mediated immunity, all of which may contribute to protective efficacy depending on the target pathogen and vaccine design. Key immunological endpoints relevant to PAdV-based swine vaccines are summarized in Table 4.

### 6.1. Humoral Immune Responses

Humoral immunity is one of the most commonly evaluated outcomes in adenovirus-vectored swine vaccine studies. Both PAdV and non-PAdV adenoviral vectors can induce antigen-specific serum antibody responses against several swine pathogens. In the CSFV model, recombinant PAdV expressing gp55/E2 induced antigen-specific and virus-neutralizing antibodies associated with protection, providing proof-of-concept evidence that PAdV can deliver protective glycoprotein antigens and induce functional humoral immunity in pigs [19,20,21].

Neutralizing antibodies are especially important when surface glycoproteins are major protective antigens. CSFV E2-specific neutralizing antibodies are linked to protection, making PAdV-gp55/E2 a clear model of antibody-mediated protection after PAdV vaccination [19,21]. Similar principles apply to enteric coronaviruses and influenza, where adenovirus-vectored vaccines expressing PEDV spike or neutralizing epitopes, and SI hemagglutinin or nucleoprotein, induced antibody responses contributing to protection [24,28,44,45,52].

However, antibody magnitude alone does not always predict protection, as shown by ASFV antigen-cocktail studies, where strong antibodies produced inconsistent protection depending on the antigen combination, regimen, and challenge model [26,27,31,32,33,48]. Future PAdV studies should therefore combine antibody measurement with neutralization, isotype analysis, functional assays, cellular immunity, and challenge outcomes.

### 6.2. Mucosal Immune Responses

Mucosal immunity is highly relevant for swine vaccines because many important pathogens infect pigs through respiratory or enteric routes, including PEDV, TGEV, SIV, PRRSV, and other respiratory pathogens. Adenoviral vectors are attractive for mucosal vaccination because they can infect epithelial or antigen-presenting cells and stimulate local immune activation. Early PAdV studies evaluated recombinant PAdV expressing CSFV gp55/E2 by oral and subcutaneous routes, demonstrating the feasibility of mucosal or enteric exposure to PAdV-based vectors, although immune responses and protection varied by route [20,21].

The TGEV model is particularly relevant because recombinant PAdV-5 expressing the TGEV spike showed that PAdV-5 can carry and express a major coronavirus antigen [16]. Since TGEV and PEDV are enteric coronaviruses, future PAdV-based coronavirus vaccines should prioritize intestinal IgA, neutralizing antibodies, and lactogenic immunity. Adenovirus-vectored PEDV studies expressing spike or neutralizing epitopes further support mucosal vaccine design [28,52].

For respiratory diseases, intranasal or aerosol adenoviral delivery may induce local respiratory immunity. Adenoviral-vectored SI vaccines have protected pigs and modern epigraph or epitope-optimized constructs have induced durable immunity [24,44,45,47]. However, direct studies of PAdV-induced nasal IgA, bronchial immunity, and local T-cell responses remain limited.

### 6.3. Cellular Immune Responses

Cell-mediated immunity is a major advantage of adenoviral vector vaccines because intracellular antigen expression enables presentation through major histocompatibility complex class I and II pathways, stimulating CD8+ cytotoxic T cells, CD4+ helper T cells, and IFN-γ production. These responses are important for intracellular viral infections and pathogens for which antibodies alone are insufficient.

In pigs, cellular immunity has been most clearly emphasized in adenovirus-vectored vaccines for ASFV, PRRSV, and SI. For ASFV, adenovirus-vectored antigen cocktails induced IFN-γ responses and cytotoxic T-cell activity, although protection varied, highlighting the need to identify protective T-cell antigens and immune correlates [26,27,29,31,33]. PRRSV adenoviral vectors expressing GP3, GP5, M protein, or antigen combinations induced antigen-specific cellular responses and reduced viral load in some models, supporting evaluation by IFN-γ ELISpot, lymphocyte proliferation, cytokine profiling, and CD4+/CD8+ phenotyping [25,30,42,43]. For SI, adenoviral vectors expressing hemagglutinin and nucleoprotein protected pigs and helped overcome maternal antibody interference, while epigraph approaches may broaden cellular and humoral immunity [24,44,46,47]. Therefore, cellular immunity should be a core endpoint in future PAdV vaccine development.

### 6.4. Innate Immune Activation and Adjuvant-like Effects

Adenoviral vectors possess intrinsic immunostimulatory properties. Viral particles, capsid proteins, and vector DNA can activate innate immune pathways, leading to cytokine and chemokine production, dendritic cell activation, and enhanced antigen presentation. These innate responses contribute to the adjuvant-like activity of adenoviral vectors and help explain their ability to induce strong adaptive immunity without conventional adjuvants [5,6,7]. In pigs, innate activation may support vaccine efficacy, but excessive inflammation could affect safety, reactogenicity, or vector clearance.

Some adenovirus-vectored swine vaccine studies have used immunomodulatory molecules to enhance responses. For example, adenovirus-mediated CD40 ligand expression improved the immunogenicity of PRRSV GP3 and GP5 antigens in swine, showing that adenoviral vectors can deliver both pathogen antigens and immune-enhancing signals [25]. Similar approaches could be applied to PAdV vectors by incorporating cytokines, co-stimulatory molecules, chemokines, or molecular adjuvant domains. However, these designs require careful safety and regulatory evaluation because immunomodulatory inserts may alter tissue responses or reactogenicity. Future PAdV studies should systematically assess type I interferon responses, inflammatory cytokines, dendritic cell activation, macrophage responses, and mucosal immune signaling to determine whether additional adjuvants or vector modifications are needed.

### 6.5. Maternal Antibody Interference and Vaccination of Young Pigs

Maternal antibody interference is a major practical challenge for swine vaccination. Piglets often receive maternal antibodies through colostrum, and these antibodies can reduce the effectiveness of conventional vaccines by neutralizing vaccine antigens or preventing adequate immune priming. Adenoviral vectors may help overcome this limitation because they deliver antigen genes into host cells and can stimulate both humoral and cellular immunity. Early work with replication-defective adenovirus-vectored AD vaccines showed that piglets vaccinated at birth in the presence of maternal antibodies developed high antibody levels and protection against AD challenge [54]. This finding is important because it suggests that adenoviral vectors may be useful for early-life immunization.

SI studies also support this concept. Recombinant HAdV-5 vectors expressing SI hemagglutinin and nucleoprotein helped overcome maternal antibody interference and induced protective immunity in young pigs [46]. These findings are highly relevant for PAdV-based vaccine development because many swine vaccines must be administered early in life. If PAdV vectors can induce immune responses despite maternal antibodies, they may provide practical advantages for piglet vaccination programs. However, this potential must be tested directly because maternal antibodies against PAdV itself, pathogen antigens, or both could influence vaccine performance.

### 6.6. Route-Dependent Immune Outcomes

The route of adenoviral vector administration strongly influences immune outcome. Parenteral vaccination, such as intramuscular or subcutaneous injection, generally favors systemic antibody and cellular immune responses. Mucosal vaccination, including oral or intranasal administration, may induce local IgA and tissue-resident immune responses, which are important for enteric and respiratory pathogens. Early PAdV studies showed that oral and subcutaneous vaccination with recombinant PAdV expressing CSFV gp55/E2 produced different immune outcomes, indicating that route optimization is essential [20,21].

For enteric pathogens such as TGEV and PEDV, oral or other mucosal routes may be desirable, but they also present challenges related to vector stability in the gastrointestinal tract, uptake by mucosal lymphoid tissue, induction of tolerance, and dosing consistency. For respiratory pathogens such as SI and PRRSV, intranasal delivery may improve local protection but requires evaluation of vector safety, shedding, and respiratory tract inflammation. Therefore, future PAdV studies should compare routes systematically using standardized immune endpoints, including serum IgG, neutralizing antibodies, mucosal IgA, IFN-γ responses, tissue-specific immune cells, and challenge outcomes.

### 6.7. Correlates of Protection and Interpretation of Immune Outcomes

One of the major challenges in interpreting adenovirus-vectored vaccine studies in pigs is the lack of consistent correlates of protection across diseases. For CSFV, neutralizing antibodies against E2 are closely associated with protection, and this helps explain the success of the recombinant PAdV-gp55/E2 studies [19,21]. For SI, hemagglutination inhibition antibodies, neutralizing antibodies, mucosal immunity, and T-cell responses may all contribute to protection. For PEDV and TGEV, intestinal and lactogenic immunity are critical. For PRRSV and ASFV, protective immunity is more complex and may require coordinated antibody and cellular responses.

These disease-specific differences mean that PAdV vaccine development should not rely on a single immune endpoint. Instead, immune evaluation should be tailored to the target pathogen. For enteric diseases, mucosal IgA, neutralizing antibodies, and sow-to-piglet lactogenic immunity should be emphasized. For respiratory diseases, nasal IgA, lung T-cell responses, viral shedding, and lesion reduction should be included. For systemic diseases such as ASFV and CSFV, neutralizing antibodies, IFN-γ responses, cytotoxic T-cell activity, viremia reduction, clinical protection, and survival should be evaluated. Such pathogen-specific immune profiling will be essential for determining whether PAdV vectors can provide meaningful protection.

### 6.8. Implications for Future PAdV-Based Vaccine Development

The immunological evidence suggests that PAdV-based platforms may support systemic antibody responses, cellular immunity, and mucosal delivery, but direct PAdV data remain narrow and are largely derived from CSFV, PRV, and TGEV studies. Therefore, immune outcomes observed with other adenoviral vectors should be treated as comparative guidance rather than direct evidence of PAdV performance.

Future PAdV studies should include integrated immune profiling rather than serum antibody measurement alone. Minimum endpoints should include antigen-specific IgG, neutralizing antibodies, mucosal IgA when relevant, IFN-γ responses, T-cell subsets, cytokine profiles, the duration of immunity, anti-vector immunity, and challenge outcomes. These endpoints should be selected according to the target disease and interpreted together with protection, shedding, and safety data.

In summary, PAdV immunological potential remains promising but incomplete. The field now requires standardized, disease-specific immune evaluation to determine whether PAdV vectors can provide meaningful advantages as next-generation swine vaccine platforms.

## 7. Potential Strategic Advantages of PAdV as a Swine Vaccine Vector

PAdV-based vectors offer potential strategic advantages for swine vaccine development, but most remain only partially validated experimentally. The strongest rationale is porcine origin: PAdV is naturally associated with pigs and may provide a host-relevant backbone for swine antigen delivery, mucosal interaction, and target-animal applicability [34,35]. Early studies demonstrate that PAdV can express heterologous antigens from CSFV, PRV, and TGEV and induce immune responses in pigs [16,19,20,21,22,23].

A second potential advantage is engineering flexibility. PAdV vectors may be designed as replication-defective, replication-competent, attenuated, or prime-boost components depending on the target disease, delivery route, safety requirement, and desired immune profile [15,22]. This flexibility could support systemic vaccination, mucosal delivery, DIVA-compatible constructs, modular antigen replacement, and multivalent vaccine design, but these applications should be regarded as development opportunities rather than established field advantages.

The practical value of PAdV will depend on whether these theoretical advantages translate into competitive immunogenicity, safety, manufacturability, anti-vector immunity profiles, and protection in target animals. These features provide a rationale for further PAdV development, but they should not be interpreted as proven field advantages. At present, most proposed advantages remain only partially validated and require direct testing in target animals. The comparative value of PAdV relative to other adenoviral platforms is discussed separately in Section 9.

## 8. Challenges, Biosafety, and Regulatory Considerations

Although PAdV-based vectors have several attractive features for swine vaccine development, important challenges must be addressed before they can be translated into practical veterinary vaccines. These challenges include the limited number of direct PAdV vaccine studies, incomplete knowledge of anti-vector immunity in pig populations, uncertainty regarding optimal vector design, insufficiently developed producer cell systems, and limited data on safety, shedding, recombination, and large-scale manufacturing. Because PAdV vectors are intended for use in livestock populations, biosafety and regulatory requirements are particularly important. A successful PAdV-based vaccine platform must therefore demonstrate not only immunogenicity and protection but also genetic stability, target-animal safety, production consistency, and environmental safety. The major translational challenges and future research priorities for PAdV vaccine development are summarized in Table 5.

### 8.1. Limited Evidence Base and Lack of Modern Target-Animal Studies

The most immediate limitation of PAdV-based vaccine development is the small number of direct vaccine studies. Most experimental PAdV vaccine studies were conducted more than two decades ago and focused mainly on CSFV, PRV, and TGEV [16,19,20,21,22]. These studies provided important proof-of-concept evidence, but they were performed using early-generation vector systems and relatively limited immunological tools. Modern analyses of cellular immunity, mucosal immunity, innate immune activation, antigen-specific T-cell subsets, transcriptomic responses, and systems vaccinology were generally not included.

In addition, direct studies evaluating PAdV vectors against contemporary high-impact swine pathogens remain scarce. Diseases such as ASF, PRRS, PED, and SI represent major current priorities for swine vaccine development, but PAdV-based vaccines have not yet been systematically evaluated against these pathogens using modern vector designs. This evidence gap limits the ability to judge whether the advantages of PAdV are superior to, or even comparable with, other adenoviral platforms. Therefore, renewed target-animal studies are required to evaluate optimized PAdV vectors in pigs under standardized experimental conditions.

### 8.2. Pre-Existing Immunity to PAdV in Pig Populations

Pre-existing immunity is a major concern for adenoviral vector platforms. One rationale for using non-HAdV is to avoid immunity against common HAdV [9,10,60]. However, for PAdV-based vaccines in pigs, the key issue is pre-existing immunity to PAdV circulating in swine populations. Neutralizing antibodies or cellular immunity against the selected PAdV serotype could reduce vector transduction, antigen expression, and vaccine efficacy.

This issue remains poorly characterized. Molecular studies have detected PAdV-5 in diarrheic pigs, indicating that field exposure occurs in swine populations [17]. However, comprehensive seroepidemiological data are limited, and the prevalence of neutralizing antibodies against different PAdV serotypes in commercial herds remains unclear. Before PAdV vectors can advance toward commercial use, studies should determine the prevalence, magnitude, and cross-reactivity of anti-PAdV antibodies in pigs from different ages, production systems, and geographic regions.

Anti-vector immunity may also affect repeated vaccination because booster doses are common in swine vaccine programs. Immunity induced after the first PAdV dose could reduce responses to subsequent doses using the same vector. Therefore, heterologous prime-boost strategies, alternative PAdV serotypes, low-seroprevalence serotype selection, capsid modification, alternative administration routes, or heterologous vector combinations may be needed, but these approaches require experimental validation.

### 8.3. Vector Shedding, Transmission, and Environmental Safety

Because livestock vaccines may be used in large animal populations, vector shedding and environmental dissemination are important biosafety considerations. Recombinant viral vector vaccines often require environmental risk assessment because they may be classified as genetically modified organisms. Evaluation should consider replication competence, host range, tissue tropism, shedding, persistence, transmission potential, and environmental exposure [61,62,63]. Replication-competent PAdV vectors may enhance antigen expression and mucosal stimulation, but they also raise concerns about shedding in feces, respiratory secretions, oral fluids, or other biological materials. This is especially relevant for oral or intranasal vaccination, where local replication, persistence, and route-dependent excretion may occur [34,64].

Replication-defective PAdV vectors may reduce shedding and transmission risks, but they still require evaluation because vector particles may be released after vaccination. Biodistribution and shedding can vary according to vector type, dose, host species, and administration route [64,65,66]. Future studies should therefore include molecular and infectivity-based assays using nasal swabs, oral fluids, feces, blood, and relevant tissues. Farm-level exposure should also be assessed, including possible spread to contact pigs and clinical, virological, or immunological consequences. These data will support safe use conditions, environmental risk assessment, and regulatory evaluation of PAdV-based veterinary vaccines [61,62,67].

### 8.4. Recombination and Genetic Stability

Recombination and genetic stability are key biosafety and quality concerns for recombinant PAdV vectors. In pigs previously exposed to circulating field PAdV strains, co-infection between a recombinant vector and field virus could theoretically allow homologous recombination, leading to restoration of deleted genes, loss of the transgene, altered tropism, or emergence of recombinant viruses with unpredictable properties. Similar risks have been reported in adenoviral systems, particularly when homologous adenoviral sequences are present in producer cells or when replication-competent adenovirus arises during propagation [68,69,70,71]. Although the actual risk for PAdV remains uncertain, it should be addressed during vector design, producer cell selection, in vivo safety testing, and regulatory evaluation [34,61,62].

Genetic stability is equally important because PAdV vectors must retain inserted antigen genes during production, storage, and in vivo use. Large antigens or multigene cassettes may reduce vector fitness and be deleted during serial passage. Adenovirus studies show that genome size, insertion site, and packaging capacity affect vector stability [41,72]. In PAdV, the functional role of the E3-encoded 13.7 K structural protein and recent PAdV-5 reverse genetics emphasize the need to define essential regions experimentally [18,39]. Candidate PAdV vaccines should therefore undergo serial-passage testing, full-genome sequencing, restriction analysis, antigen-expression assays, infectious-titer measurement, and testing for revertants or helper-virus contamination, consistent with recombinant vector quality expectations [61,62,73].

### 8.5. Producer Cell Lines and Manufacturing Scalability

Manufacturing remains a major barrier to PAdV vaccine translation. HAdV platforms benefit from established producer cell lines, scalable production, purification methods, and regulatory experience, whereas PAdV systems are less mature [8,74,75,76,77]. Replication-competent PAdV vectors may be produced in permissive porcine cells, but replication-defective vectors require complementing cell lines that supply deleted viral functions. Although E1-deleted PAdV-3 vectors have been generated, stable, high-yield, regulatory-acceptable producer systems for commercial PAdV vaccines remain insufficiently developed [5,15,34].

An ideal PAdV production system should support efficient vector rescue, high-titer amplification, genetic stability, purity, scalability, and batch consistency. Compatibility with serum-free or chemically defined media, suspension culture, bioreactors, nuclease treatment, chromatography-based purification, and stable formulation will be important for product quality [75,78,79]. Regulatory expectations also include cell-substrate characterization, adventitious-agent testing, identity and purity testing, cell-bank stability, and absence of replication-competent or helper-virus contaminants [62,73,80]. Because swine vaccines must be low cost and scalable, future PAdV development should evaluate production yield, purification feasibility, formulation stability, storage needs, dose sparing, and GMP compatibility from the early stage [34,62,75,79].

### 8.6. Safety in Target Animals

Target-animal safety is essential for veterinary vaccine approval. PAdV vectors must be evaluated in pigs of different ages, physiological states, and immune backgrounds. Safety assessment should include local and systemic reactions, fever, weight gain, feed intake, clinical signs, hematological and biochemical parameters, and gross or histopathological lesions when appropriate. These endpoints are consistent with safety expectations for veterinary vaccines and live recombinant vector vaccines [62,81]. For breeding animals, reproductive safety may be required, while piglet vaccines should assess neonatal safety and the effects of maternal antibodies on both safety interpretation and immune efficacy [1,46,50].

Safety requirements may differ between replication-competent and replication-defective PAdV vectors. Replication-competent vectors require detailed assessment of shedding, tissue distribution, reversion, recombination, and spread to contact animals, whereas replication-defective vectors still require evaluation of inflammation, biodistribution, persistence, and particle release [61,62,65]. Route-specific safety is also important because oral or intranasal delivery may increase mucosal, environmental, and contact-animal exposure. Monitoring should include clinical observation and vector detection in nasal swabs, oral fluids, feces, blood, and selected tissues [21,23,34,82]. Later-stage studies should use representative field conditions, including concurrent infections, stress, immunosuppression, variable microbiota, pre-existing immunity, growth performance, shedding, contact transmission, immune responses, and compatibility with existing vaccination programs [62,81,82].

### 8.7. DIVA Compatibility and Diagnostic Interference

Differentiation of infected from vaccinated animals (DIVA) is important for transboundary and regulated swine diseases, including CSF, AD, and ASF. DIVA-compatible vaccines support surveillance, outbreak control, and international trade by enabling diagnostic tests to distinguish naturally infected animals from vaccinated animals [55,56,58,83]. PAdV vectors may support DIVA-oriented vaccine design because they can express selected protective antigens rather than the whole pathogen. This approach could allow companion diagnostics to detect antibodies against non-vaccine antigens. Similar principles have been used in CSF E2-based marker vaccines and AD gE-deleted vaccines combined with gE-based serological assays [55,56,58,83,84].

However, DIVA compatibility must be integrated from the beginning. Antigen selection, diagnostic marker availability, and companion test performance should be developed together. For CSF, an E2-expressing PAdV vaccine could theoretically be paired with Erns- or NS-based diagnostic assays, although field sensitivity and specificity would require validation [55,56,57,58]. For AD, gE-deleted marker systems provide a strong precedent for DIVA-based eradication programs [83,84,85]. For ASF, DIVA design remains more difficult because protective antigens, correlates of protection, vaccine safety, and companion diagnostics are still being defined [2,4,86]. Therefore, future PAdV vaccines should integrate DIVA strategies, companion diagnostics, antigen selection, and regulatory requirements early in vector design.

### 8.8. Regulatory Considerations for Genetically Modified Viral Vector Vaccines

PAdV-based vaccines would likely be regulated as recombinant viral vector vaccines or genetically modified biological products. Although requirements may vary among countries, common expectations include clear documentation of vector construction, genetic stability, identity, purity, potency, target-animal safety, efficacy, and environmental risk assessment. Live recombinant vector vaccines require information on the vector, inserted gene, genetic modification strategy, biological properties, safety, efficacy, and environmental exposure [62]. Because recombinant viral vectors may be considered genetically modified organisms, risk assessment should address potential effects on animals, humans, and the environment [61]. For replication-defective vectors, regulators may require proof that the vector cannot replicate in normal host cells and that replication-competent revertants or helper-virus contaminants are absent. Replication-competent vectors require broader evaluation of shedding, transmission, reversion, recombination, tissue distribution, host range, and environmental persistence [62,81,82].

Regulatory review will also consider the inserted antigen, promoter, deletion strategy, cell substrate, production process, residual host-cell DNA, adventitious agent testing, and quality-control assays. Cell substrates must be well characterized because origin, growth properties, adventitious agents, genetic stability, and tumorigenicity may affect safety and quality [73,87]. For food-producing animals, withdrawal periods, tissue distribution, vector persistence, and food safety may also be relevant. Because no commercial PAdV-vectored vaccine is currently available, early regulatory dialogue, standardized safety packages, defined environmental risk assessment, well-characterized vector backbones, and producer cell banks will be essential to reduce translational risk and support future commercial development [62,81,82,88].

### 8.9. Integrated Translational Gaps

The central translational gap for PAdV is the lack of integrated data linking vector biology, engineering, immunogenicity, safety, manufacturing, and field applicability. Early studies established feasibility, but modern development requires coordinated evaluation of PAdV-3 and PAdV-5 backbones, seroprevalence in commercial herds, stable deletion mutants, producer cell systems, mucosal delivery, shedding, recombination risk, and target-animal efficacy.

Future studies should compare candidate PAdV vectors with existing vaccines or established adenoviral platforms using the same antigen, dose, route, and challenge model. Commercial translation will also require early assessment of manufacturability, formulation stability, the cost of goods, scalability, and regulatory readiness. These challenges are summarized in Table 5 and are converted into specific development priorities in Section 10.

## 9. Comparative Positioning with Other Adenoviral Vector Platforms

PAdV should be evaluated within the broader adenoviral-vector landscape rather than in isolation. Unlike Section 7, which summarizes the rationale for considering PAdV as a swine-associated vector, this section evaluates PAdV comparatively against established human, non-human, and animal adenoviral platforms to clarify its current translational position. HAdV-5 remains the most technically mature platform because of established genome engineering, producer cell lines, purification systems, and regulatory experience, and it has been used widely in experimental swine vaccine studies [24,25,26,28,30,77]. Its limitations for swine vaccination include anti-vector immunity after repeated use and limited target-host association. Rare HAdV and ChAd platforms offer important lessons in serotype selection and translational development, but they remain human or primate derived rather than swine associated.

Animal adenoviruses provide more relevant veterinary comparators. BAdV is informative because it is a livestock-associated adenoviral platform with broader development than PAdV and evidence for vaccine delivery and evasion of HAdV immunity [5,12]. CAdV-2 has induced PRRSV-specific immune responses in pigs but is not naturally porcine, and FAdV illustrates how host-associated adenoviruses can be developed for poultry rather than swine applications. These comparisons emphasize that host origin is valuable only if it translates into measurable improvements in safety, immune outcomes, delivery, manufacturing, or field practicality.

Compared with these platforms, PAdV has a distinct but underdeveloped position. Its strengths include porcine origin, demonstrated heterologous antigen expression, potential mucosal relevance, and flexible replication-competent or replication-defective design [14,15,18,34]. Its weaknesses include fewer studies, immature producer cell systems, limited modern target-animal efficacy data, uncertain anti-PAdV immunity, and no commercial precedent. Direct head-to-head studies are therefore essential to define whether PAdV provides advantages beyond biological plausibility.

The potential advantage of PAdV over other adenoviral platforms therefore lies not in general adenoviral immunogenicity, which is shared by many vectors, but in whether a porcine-associated backbone can provide measurable benefits in pigs. These benefits may include improved target-host compatibility, route-specific mucosal performance, reduced interference from anti-HAdV immunity, or better integration into swine-specific vaccine programs. At present, these remain testable hypotheses rather than established advantages. Direct head-to-head comparisons using the same antigen, dose, route, and challenge model are required to determine whether PAdV offers practical benefits over HAdV, ChAd, BAdV, CAdV, or other vector systems.

## 10. Future Perspectives and Translational Roadmap

**Figure 5:**
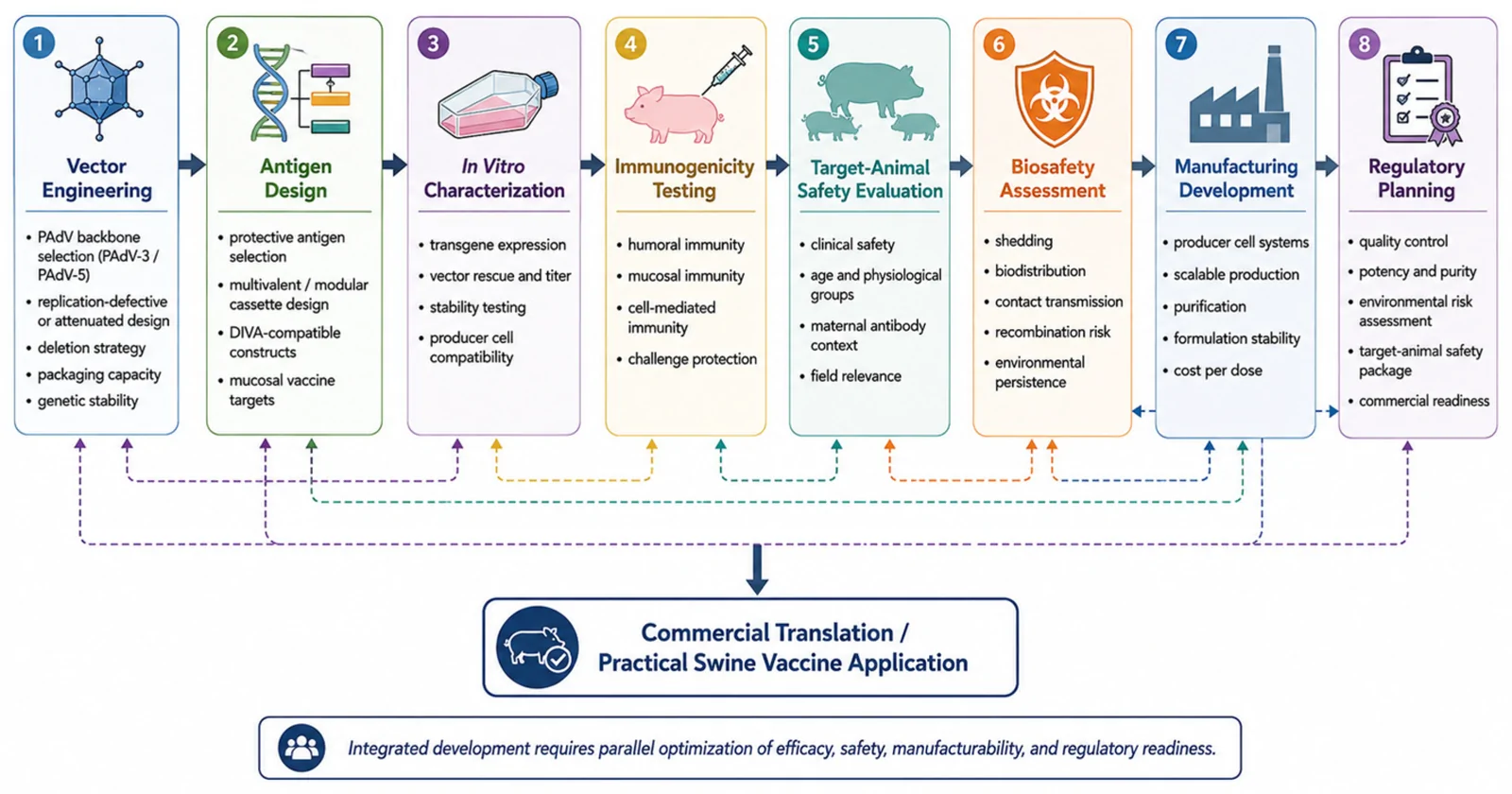
Translational roadmap for future PAdV-based swine vaccine development. This figure presents an integrated pathway for moving PAdV vectors from proof-of-concept studies toward commercial application. The roadmap includes vector-backbone selection, antigen-cassette design, in vitro characterization, immunogenicity testing, target-animal challenge studies, biosafety assessment, manufacturing optimization, and regulatory evaluation. It highlights the need to develop efficacy, safety, manufacturability, and regulatory readiness in parallel.

PAdV-based vectors remain at an early stage, but advances in reverse genetics, adenoviral vector engineering, and veterinary vaccinology provide an opportunity to revisit PAdV as a next-generation swine vaccine platform. Future progress should shift from isolated proof-of-concept studies toward systematic platform development that integrates vector engineering, antigen design, immunogenicity testing, target-animal safety, biosafety assessment, manufacturing development, and regulatory planning. The proposed translational roadmap is summarized in Figure 5.

Based on the current evidence, the most immediate research priorities are (i) direct comparison of PAdV-3 and PAdV-5 using identical antigen cassettes; (ii) definition of stable insertion sites and packaging limits for each backbone; (iii) seroprevalence and neutralization studies of anti-PAdV immunity in commercial pigs; (iv) standardized target-animal immunogenicity and challenge studies using modern immune endpoints; (v) shedding, biodistribution, and recombination-risk assessment for replication-competent and replication-defective vectors; and (vi) development of scalable producer cell systems and quality-control assays. These priorities are derived from the current limitations of the evidence base rather than from general platform expectations.

### 10.1. Modern Vector Engineering and Backbone Selection

Future PAdV development should prioritize efficient reverse genetic systems, standardized PAdV-3 and PAdV-5 backbones, and experimentally validated deletion or insertion sites [14,15,18]. Comparative characterization should include growth kinetics, transgene capacity, antigen expression, genetic stability, receptor usage, tissue tropism, anti-vector immunity, and safety. Replication-defective E1- or E1/E3-deleted vectors may be preferred for commercial livestock applications because they reduce concerns about uncontrolled replication, transmission, and environmental spread, but they will require stable complementing producer cell lines and rigorous testing for replication-competent revertants or helper-virus contaminants [15,62,68,69,70,73]. Replication-competent, single-cycle, or attenuated PAdV vectors may still be useful for mucosal vaccination if shedding, recombination, biodistribution, and environmental persistence can be controlled [7,34,61,62,89].

### 10.2. Disease-Specific Antigen and Mucosal Vaccine Design

The success of PAdV vaccines will depend on rational antigen design rather than vector selection alone. For CSFV, optimized E2 expression and DIVA-compatible strategies remain logical. For PEDV and TGEV, spike protein or conserved neutralizing epitopes should be paired with mucosal delivery to promote intestinal IgA, neutralizing antibodies, and lactogenic immunity. For PRRSV, multivalent constructs expressing GP5, M, GP3, conserved T-cell antigens, or immune modulators may be required. For SIV, epigraph, consensus, or conserved-antigen approaches could broaden protection. For ASFV, multigene antigen combinations and cellular immune endpoints should be prioritized because single antigens are unlikely to provide robust protection [1,3,30,32,33,44]. Future studies should compare oral, intranasal, aerosol, intramuscular, and combined routes using standardized readouts such as serum IgG, neutralizing antibodies, mucosal IgA, cytokine profiles, tissue-resident T cells, viral loads, shedding, lesion reduction, and clinical protection.

### 10.3. Modular, Multivalent, DIVA-Compatible, and Prime-Boost Platforms

**Figure 6:**
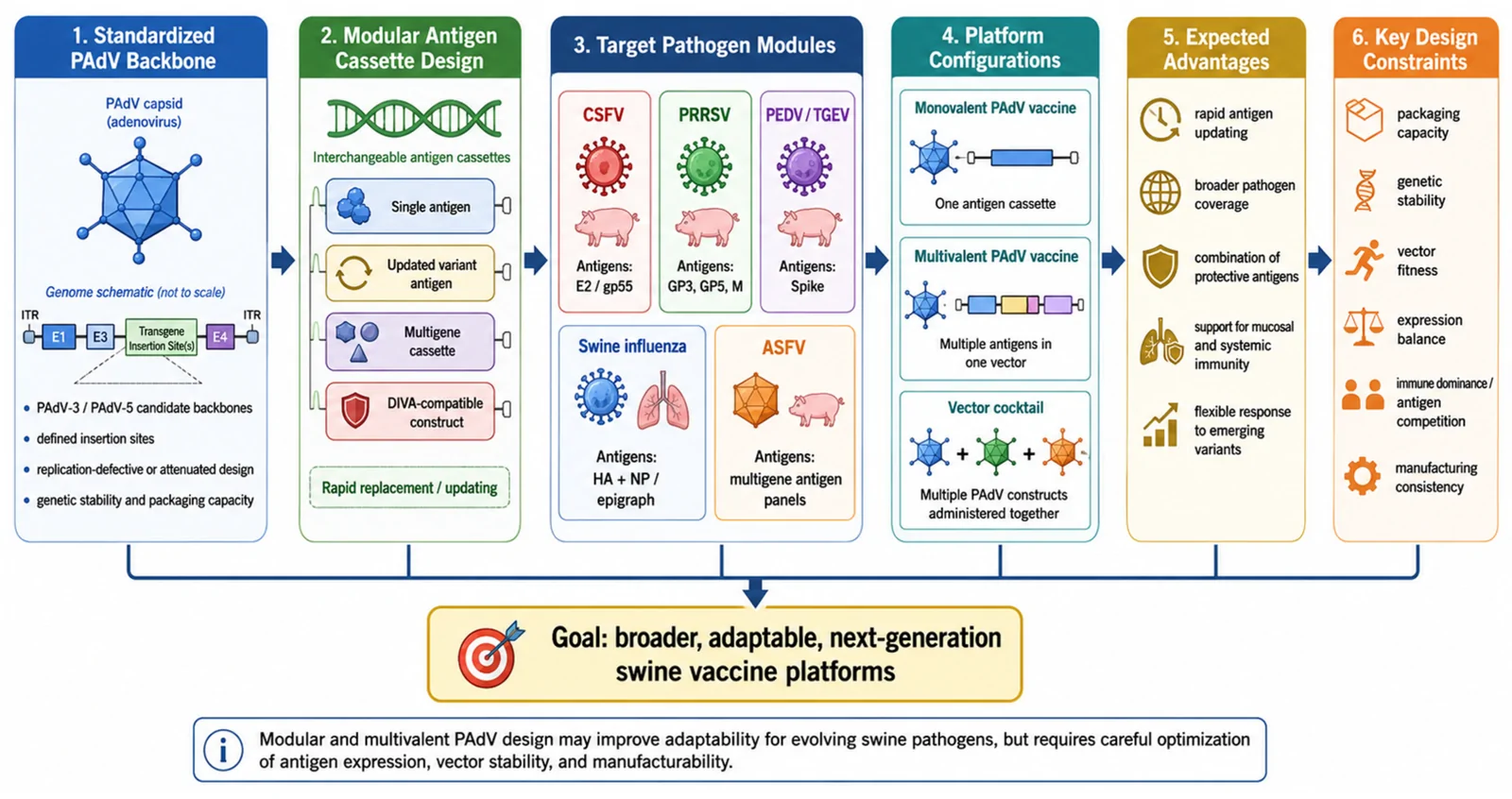
Proposed design strategy for modular and multivalent PAdV vaccine platforms. This figure illustrates how a standardized PAdV backbone could express interchangeable antigen cassettes for different pathogens or emerging variants. Potential antigen modules include CSFV E2/gp55, PRRSV GP3/GP5/M, PEDV/TGEV spike, SIV hemagglutinin and nucleoprotein, and ASFV multigene antigen panels. The figure also highlights key constraints, including antigen-expression balance, immune dominance, genetic stability, packaging capacity, vector fitness, and manufacturing consistency.

Commercial swine production would benefit from vaccine platforms that can be rapidly updated for emerging variants or combined antigenically complex pathogens. PAdV vectors could be developed as modular platforms using standardized backbones and interchangeable antigen cassettes. Multivalent strategies may include multiple antigens within one vector or cocktails of different PAdV constructs, but these designs require assessment of packaging capacity, genetic stability, antigen competition, expression balance, immune dominance, vector fitness, and serial-passage stability [6,8,24,25,26,27,28,29,30,31,44,47,48,77]. The potential use of PAdV vectors as modular and multivalent vaccine platforms is shown in Figure 6.

DIVA compatibility and prime-boost strategies should be integrated early in PAdV design. Because PAdV can express selected antigens rather than whole pathogens, it may support marker-vaccine approaches for CSF, AD, and ASF when paired with companion diagnostics [55,56,57,58,83,84,85,86]. PAdV may also function in heterologous regimens, as DNA priming followed by recombinant PAdV boosting protected pigs against CSF [22]. Future studies should optimize antigen order, dose intervals, delivery routes, anti-vector immunity, and compatibility with commercial herd management [6,8,9,10,11,21,22,77].

### 10.4. Manufacturing, Biosafety, and Regulatory Readiness

Scalable production is essential for commercial translation. PAdV development should include stable producer cell lines for replication-defective vectors, optimized permissive cells for replication-competent vectors, serum-free suspension culture, high-titer amplification, chromatography-compatible purification, formulation stability, cost-per-dose evaluation, and GMP compatibility [8,62,73,75,76,77,78,79,82]. Formulation strategies such as lyophilized, spray-dried, thermostabilized, or excipient-optimized products may improve field usability and reduce cold-chain dependence.

Biosafety and regulatory planning should be incorporated from the beginning. Key studies should evaluate replication in normal pig cells, shedding, biodistribution, tissue persistence, contact transmission, recombination with field PAdV strains, environmental persistence, target-animal safety, and absence of adventitious agents or replication-competent contaminants [34,61,62,64,65,66,67,68,69,70,81,82]. Farm-level studies should include relevant samples such as nasal swabs, oral fluids, feces, blood, and tissues. Because no commercial PAdV-vectored vaccine is currently available, early regulatory dialogue, well-characterized vector backbones, standardized quality-control assays, and defined environmental-risk packages will be needed to reduce development risk [62,73,82].

### 10.5. Head-to-Head Comparison and Commercial Translation

A critical future step is direct comparison of PAdV with established adenoviral platforms such as HAdV-5, ChAd, BAdV, or CAdV using the same antigen, dose, route, and challenge model. Outcomes should include antigen expression, humoral immunity, cellular immunity, mucosal immunity, anti-vector immunity, safety, shedding, manufacturability, and protection. These comparisons will determine whether PAdV provides measurable advantages in target-host compatibility, mucosal responses, reduced vector interference, or field applicability. If PAdV does not outperform established platforms immunologically, its value may still depend on swine-specific suitability, production cost, regulatory acceptance, or integration into modular veterinary vaccine programs. Overall, future research must convert PAdV from a biologically plausible concept into a standardized, scalable, and evidence-supported swine vaccine.

## 11. Limitations of This Scoping Review

This scoping review has several limitations. First, the direct PAdV vaccine evidence is limited in number and is dominated by early proof-of-concept studies involving CSFV, PRV, and TGEV. Second, the broader adenovirus-vectored swine vaccine literature is heterogeneous in vector backbone, antigen, route, animal model, immune endpoints, dose, and challenge design, preventing quantitative pooling or formal meta-analysis. Third, some conclusions rely on comparative evidence from non-PAdV adenoviral platforms; these comparisons are useful for identifying design principles but should not be interpreted as direct validation of PAdV efficacy.

This review therefore emphasizes evidence mapping and critical interpretation rather than quantitative effect estimation. Future updates should incorporate standardized search documentation, clearer separation of primary and review evidence, and new target-animal studies that directly compare optimized PAdV vectors with established vaccine platforms.

Despite these limitations, the present review differs from earlier PAdV and nonhuman adenovirus reviews by explicitly mapping the evidence level of each study group, distinguishing direct PAdV vaccination evidence from comparative adenoviral-vector evidence, and incorporating recent PAdV-5 reverse-genetics advances into a translational framework for swine vaccine development.

## 12. Conclusions

PAdV-based vectors represent a promising but still underdeveloped platform for swine vaccine development. Current evidence shows that PAdV can be genetically manipulated, express heterologous antigens, and induce immune responses or protection in selected pig models. Early studies using CSFV, PRV, and TGEV antigens established proof-of-concept evidence, while recent PAdV-5 isolation and reverse genetics have renewed interest in the platform.

The main rationale for PAdV lies in its porcine origin, antigen-expression capacity, potential mucosal relevance, and engineering flexibility. However, the practical value of this platform remains unproven and will depend on direct evidence for target-animal efficacy, biosafety, anti-vector immunity, genetic stability, scalable production, DIVA compatibility, and regulatory feasibility. PAdV should therefore be considered an emerging swine-specific adenoviral vector rather than a mature vaccine technology.

Future work should prioritize comparative characterization of PAdV-3 and PAdV-5, stable replication-defective backbones and producer cell lines, standardized immune and challenge models, mucosal and multivalent designs, and head-to-head comparisons with established adenoviral platforms. Addressing these priorities will determine whether PAdV can become a useful tool for next-generation swine vaccine development and veterinary disease control.

## Figures and Tables

**Table 1:** Main included study groups and their roles in the scoping synthesis.

Evidence Category	Main Study Group	Role in This Review
PAdV biology and genome characterization	PAdV-3 genome map, PAdV-5 molecular characterization, PAdV-4 fiber structure	Defines genome organization, candidate serotypes, capsid features, and vector-relevant biology
PAdV vector engineering	PAdV-3 expression vector, E1-deleted PAdV-3, E3-deleted PAdV-5, rapid recombinant PAdV generation, PAdV-5 reverse genetics	Defines feasibility of recombinant vector construction and genetic manipulation
Direct PAdV-based vaccine studies	CSFV gp55/E2, PRV-gD, TGEV spike, DNA prime–PAdV boost	Provides direct proof-of-concept evidence for PAdV antigen delivery and vaccination in pigs
Comparative adenovirus-vectored swine vaccine studies	Adenoviral vectors for PRRSV, PEDV, SIV, ASFV, and PRV models	Provides disease-specific design lessons but not direct proof of PAdV efficacy
Immunological endpoint studies	Humoral, mucosal, cellular, innate, lactogenic, and challenge-protection outcomes	Defines immune endpoints for future PAdV studies
Translational and regulatory evidence	Anti-vector immunity, genetic stability, shedding, manufacturing, producer cells, biosafety, DIVA, regulatory guidance	Defines barriers and priorities for platform translation

**Table 2:** Representative direct porcine adenovirus-based vaccine studies.

Target Pathogen/Disease	Antigen or Insert	Vector Backbone/Strategy	Route/Regimen	Main Immune Outcome	Protection/Key Finding	Ref.
Classical swine fever virus (CSFV)	gp55/E2	Recombinant PAdV-3 expressing CSFV gp55/E2	Single vaccination	CSFV-specific and neutralizing antibodies	Target-animal evidence: Protected pigs against CSFV challenge; provided direct proof-of-concept for PAdV vaccination	[19]
CSFV	gp55/E2	Recombinant PAdV-gp55/E2	Oral and subcutaneous administration	Route-dependent antibody responses	Target-animal route evidence: Both routes induced immune responses, but magnitude and consistency differed by route	[23]
CSFV	gp55/E2	DNA prime followed by recombinant PAdV boost	Heterologous prime–boost	Enhanced immune response after boosting	Target-animal prime-boost evidence: Protected pigs against CSFV challenge; supported PAdV use in sequential vaccination	[22]
Pseudorabies virus (PRV)	gD	Recombinant PAdV expressing PRV gD	Experimental vaccination	Antigen-specific immune response	Target-animal immunogenicity evidence: Demonstrated that PAdV can deliver herpesvirus antigen	[20]
Transmissible gastroenteritis virus (TGEV)	Spike protein	Recombinant PAdV-5 expressing TGEV spike	Experimental expression/vaccine model	Spike antigen expression	Vector-construction evidence: Demonstrated feasibility of PAdV-5 for coronavirus spike antigen delivery	[16]
Platform development	E1-deleted vector/recombinant expression system	PAdV-3 vector engineering and complementing cells	In vitro vector construction	Heterologous gene expression	Vector-development evidence: Established molecular basis for PAdV vector development	[14,15]
Platform renewal	Reverse genetics/recombinant manipulation	PAdV-5	Reverse genetics system	Genetic manipulation feasibility	Reverse-genetics evidence: Renewed interest in PAdV-5 as a candidate swine-specific vector backbone	[18]

**Table 3:** Comparative adenoviral-vector vaccine evidence for major swine diseases.

Disease/Pathogen	Main Antigen(s)	Adenoviral Vector Platform	Main Immune Outcome	Protection/Disease Outcome	Relevance to PAdV Development	Ref.
CSFV	E2/gp55	PAdV	Neutralizing antibodies	Protection against CSFV challenge	Direct PAdV evidence: Strongest direct evidence for PAdV-based vaccination	[19,20,21,22]
PRRSV	GP3, GP5, M	HAdV and CAdV vectors	Humoral and cellular responses	Reduced viral load or improved immune responses in some models	Comparative non-PAdV evidence: Supports use of adenoviral vectors for PRRSV antigen delivery; suggests need for multivalent antigen design	[25,30,42,43]
PEDV	Spike protein, neutralizing epitopes	Recombinant adenovirus	PEDV-specific antibody and mucosal-oriented responses	Supports adenoviral delivery of coronavirus antigens	Comparative adenoviral evidence: Relevant to oral or mucosal PAdV vaccine design	[3,28]
TGEV	Spike protein	PAdV-5	Spike antigen expression	Demonstrated coronavirus spike expression by PAdV-5	Direct PAdV-5 vector evidence: Supports technical feasibility of PAdV-based coronavirus vaccines	[16]
Swine influenza virus	Hemagglutinin, nucleoprotein, epigraph antigens	Recombinant adenovirus	Antibody and cellular responses	Protection, reduced shedding, reduced lesions, broader immunity	Comparative adenoviral evidence: Supports respiratory mucosal and computational antigen design for PAdV	[24,44,45,46,47]
African swine fever virus	Multigene antigen cocktails	Recombinant adenovirus/viral-vector cocktails	Antibody, IFN-γ, cytotoxic T-cell responses	Protection variable depending on antigen combination and regimen	Comparative adenoviral evidence: Highlights need for multivalent design, cellular endpoints, and careful antigen selection	[26,27,29,31,32,33,48]

**Table 4:** Immunological outcomes relevant to PAdV-based swine vaccines.

Immune Outcome	Key Markers/Assays	Relevant Pathogen Models	Main Interpretation	Ref.
Innate immune activation	Type I interferon, IL-6, TNF-α, IL-12, dendritic cell activation, macrophage activation	General adenoviral vector studies; PRRSV immunomodulatory vector studies	Adenoviral vectors can act as adjuvant-like platforms, but excessive activation may affect safety or vector clearance	[5,6,7,25]
Humoral immunity	Antigen-specific IgG, virus-neutralizing antibodies, antibody isotypes	CSFV, PEDV, swine influenza, ASFV	Important for surface glycoprotein antigens; neutralizing activity is more informative than antibody magnitude alone	[19,20,21,24,28,44]
Mucosal immunity	Secretory IgA, nasal IgA, intestinal IgA, local respiratory or intestinal responses	PEDV, TGEV, swine influenza, PRRSV	Critical for enteric and respiratory pathogens; should be prioritized for oral or intranasal PAdV vaccines	[16,20,21,28]
Cell-mediated immunity	IFN-γ ELISpot, lymphocyte proliferation, CD4+ T cells, CD8+ T cells, cytotoxic T-cell activity	ASFV, PRRSV, swine influenza	Essential for intracellular pathogens and complex viruses where antibodies alone are insufficient	[25,26,27,29,30,31,33]
Lactogenic immunity	Colostrum IgA/IgG, milk IgA, sow-to-piglet antibody transfer	PEDV, TGEV	Particularly important for neonatal protection against enteric coronaviruses	[1,3,50,51]
Challenge protection	Survival, clinical score, viremia, viral shedding, lung lesions, intestinal lesions	CSFV, PRRSV, PEDV, swine influenza, ASFV	Final confirmation of vaccine efficacy; should be interpreted together with immune endpoints	[19,21,24,30,31]

**Table 5:** Key translational challenges and future priorities for PAdV vaccine development.

Theme	Why It Matters	Current Gap	Suggested Priority Action
Anti-vector immunity	Pre-existing or vaccine-induced antibodies may reduce transduction, antigen expression, and booster efficacy	Seroprevalence and cross-neutralization data for PAdV-3, PAdV-5, and other serotypes remain limited	Conduct large-scale serology, neutralization, cross-neutralization, and vector-transduction inhibition studies
Genetic stability and packaging capacity	Insert loss, rearrangement, or oversized genomes may reduce efficacy and manufacturing consistency	Packaging limits and long-term stability of different PAdV backbones remain incompletely defined	Perform serial-passage testing, full-genome sequencing, antigen-expression assays, and packaging-capacity mapping
Biosafety and environmental risk	Replication, shedding, transmission, recombination, and persistence may affect regulatory acceptance	Shedding, biodistribution, contact transmission, and environmental persistence data are sparse	Conduct biodistribution, shedding, infectivity, contact-animal, and environmental persistence studies
Manufacturing and producer cell systems	Poor yield, unstable production, or lack of scalable purification will block commercialization	Stable, high-yield, regulatory-acceptable producer systems are not yet mature	Develop complementing cell lines, serum-free suspension culture, scalable purification, and batch consistency testing
Target-animal safety	Safety signals may limit approval and field adoption	Limited data across age groups, breeding animals, neonates, and pigs with variable immune backgrounds	Expand GLP-compliant safety studies, dose-escalation studies, reproductive safety, and field-condition evaluations
DIVA compatibility	Surveillance and trade management require differentiation of infected from vaccinated animals	Validated DIVA strategies for PAdV-vectored vaccines are lacking	Design antigen cassettes and companion diagnostics together from the early stage
Mucosal delivery	Many swine pathogens infect through respiratory or enteric routes	PAdV-induced nasal IgA, intestinal IgA, and local T-cell responses are poorly defined	Compare oral, intranasal, aerosol, and parenteral delivery routes with mucosal immune endpoints
Modular and multivalent design	Complex pathogens require broader and more durable protection	Design rules for multivalent antigen expression, antigen competition, and immune dominance remain unclear	Develop modular backbones, evaluate multigene cassettes, and test antigen balance and stability

## Data Availability

No new data were created or analyzed in this study. Data sharing is not applicable to this article.

## Abbreviations

The following abbreviations are used in this manuscript:

AD	Aujeszky’s disease
ASF	African swine fever
ASFV	African swine fever virus
BAdV	Bovine adenovirus
CAR	Coxsackievirus and adenovirus receptor
CAdV	Canine adenovirus
CD	Cluster of differentiation
ChAdV	Chimpanzee adenovirus
COVID-19	Coronavirus disease 2019
CSF	Classical swine fever
CSFV	Classical swine fever virus
CTL	Cytotoxic T lymphocyte
DIVA	Differentiation of infected from vaccinated animals
DNA	Deoxyribonucleic acid
E1	Early region 1
E2	Envelope glycoprotein E2
E3	Early region 3
E4	Early region 4
ELISA	Enzyme-linked immunosorbent assay
EMA	European Medicines Agency
FAdV	Fowl adenovirus
GLP	Good Laboratory Practice
GMP	Good Manufacturing Practice
GP	Glycoprotein
GP3	Glycoprotein 3
GP5	Glycoprotein 5
HAdV	Human adenovirus
HAdV-5	Human adenovirus 5
IFN-γ	Interferon gamma
IgA	Immunoglobulin A
IgG	Immunoglobulin G
IL	Interleukin
IL-6	Interleukin 6
IL-12	Interleukin 12
ITR	Inverted terminal repeat
PAdV	Porcine adenovirus
PAdV-3	Porcine adenovirus 3
PAdV-4	Porcine adenovirus 4
PAdV-5	Porcine adenovirus 5
PED	Porcine epidemic diarrhea
PEDV	Porcine epidemic diarrhea virus
PRRS	Porcine reproductive and respiratory syndrome
PRRSV	Porcine reproductive and respiratory syndrome virus
PRV	Pseudorabies virus
PRV-gD	Pseudorabies virus glycoprotein D
SARS-CoV-2	Severe acute respiratory syndrome coronavirus 2
SI	Swine influenza
SIV	Swine influenza virus
TGE	Transmissible gastroenteritis
TGEV	Transmissible gastroenteritis virus
TGEV-S	Transmissible gastroenteritis virus spike protein
TNF-α	Tumor necrosis factor alpha
VICH	International Cooperation on Harmonisation of Technical Requirements for Registration of Veterinary Medicinal Products
WHO	World Health Organization
WOAH	World Organisation for Animal Health
